# Modulation of multisensory nociceptive neurons in monkey cortical area 7b and behavioral correlates

**DOI:** 10.1152/jn.00377.2023

**Published:** 2024-07-10

**Authors:** Willie K. Dong

**Affiliations:** ^1^Department of Anesthesiology and Pain Medicine, School of Medicine, University of Washington, Seattle, Washington, United States; ^2^Beckman Institute for Advanced Science and Technology, University of Illinois Urbana-Champaign, Urbana, Illinois, United States

**Keywords:** cortical inferior parietal lobule (area 7b), multisensory integration, nociceptive neurons, peripersonal and personal space, spatial and temporal alignment

## Abstract

Wide-range thermoreceptive neurons (WRT-EN) in monkey cortical area 7b that encoded innocuous and nocuous cutaneous thermal and threatening visuosensory stimulation with high fidelity were studied to identify their multisensory integrative response properties. Emphasis was given to characterizing the spatial and temporal effects of threatening visuosensory input on the thermal stimulus-response properties of these multisensory nociceptive neurons. Threatening visuosensory stimulation was most efficacious in modulating thermal evoked responses when presented as a downward (“looming”), spatially congruent, approaching and closely proximal target in relation to the somatosensory receptive field. Both temporal alignment and misalignment of spatially aligned threatening visual and thermal stimulation significantly increased mean discharge frequencies above those evoked by thermal stimulation alone, particularly at near noxious (43°C) and mildly noxious (45°C) temperatures. The enhanced multisensory discharge frequencies were equivalent to the discharge frequency evoked by overtly noxious thermal stimulation alone at 47°C (monkey pain tolerance threshold). A significant increase in behavioral mean escape frequency with shorter escape latency was evoked by multisensory stimulation at near noxious temperature (43°C), which was equivalent to that evoked by noxious stimulation alone (47°C). The remarkable concordance of elevating both neural discharge and escape frequency from a nonnociceptive and prepain level by near noxious thermal stimulation to a nociceptive and pain level by multisensory visual and near noxious thermal stimulation and integration is an elegantly designed defensive neural mechanism that in effect lowers both nociceptive response and pain thresholds to preemptively engage nocifensive behavior and, consequently, avert impending and actual injurious noxious thermal stimulation.

**NEW & NOTEWORTHY** Multisensory nociceptive neurons in cortical area 7b are engaged in integration of threatening visuosensory and a wide range of innocuous and nocuous somatosensory (thermoreceptive) inputs. The enhancement of neuronal activity and escape behavior in monkey by multisensory integration is consistent and supportive of human psychophysical studies. The spatial features of visuosensory stimulation in peripersonal space in relation to somatic stimulation in personal space are critical to multisensory integration, nociception, nocifensive behavior, and pain.

## Graphical abstract

**Figure:**
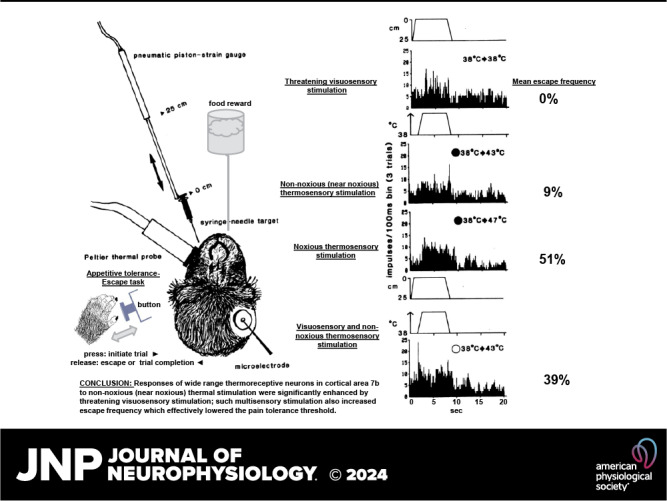

## INTRODUCTION

In a previous study of unanesthetized nonhuman primates, Dong and colleagues (1, 2) reported a subpopulation of thermal nociceptive neurons in the posterior parietal cortical area 7b (PF), which occupies the exposed convex surface area of the inferior (anterolateral) parietal lobule and the contiguous, hidden surface areas along the upper medial bank of the posterior parietal operculum (PFop), adjacent to the second somatosensory cortex (SII) (within the posterior Sylvian fissure or lateral sulcus) and along the upper lateral bank of the intraparietal sulcus, adjacent to area 5. The thermal nociceptive neurons made up 9% (21 of 244) of isolated neurons that had somatosensory response properties. Furthermore, these neurons were divided into two groups: *1*) wide-range thermoreceptive (WRT) neurons that differentially encoded a full range of innocuous and noxious thermal stimuli (WRT-EN) or did not (WRT-NE) and *2*) high-threshold thermoreceptive (HTT) neurons that encoded a full range of noxious thermal stimuli (HTT-EN) or did not (HTT-NE). Many of the WRT and HTT neurons were bimodal and responded also to innocuous and/or noxious mechanical stimulation of facial skin (see also Ref. 3). These cutaneous mechanical receptive fields on the face were often large and bilateral. The stimulus intensity-discharge frequency functions of WRT-EN and HTT-EN neurons, unlike those of WRT-NE and HTT-NE neurons, closely approximated stimulus intensity-behavioral escape frequency functions. Of particular interest, some WRT and HTT neurons (7 of 21) were multisensory (polysensory) and responded to stimulation within spatially aligned visual and cutaneous receptive fields. Threatening or novel visuosensory stimuli that approached the face along a trajectory aligned with the most sensitive portion of the cutaneous receptive field evoked the highest mean discharge frequency. Visuosensory responses were often sustained by holding the visual target close to the cutaneous receptive field. Iterative visuosensory stimulation delivered at short intervals and along the same trajectory toward the spatially aligned cutaneous receptive field produced response adaptation as observed by progressive decrements in the mean discharge frequency. The range of mean discharge frequencies in response to a range of noxious thermal stimulation (i.e., 45°C to 51°C) overlapped the range of mean discharge frequencies in response to threatening or novel visual stimulation. Within area 7b, there was no apparent somatotopic representation of the trigeminal nerve subdivisions, no visuotopic representation of visual space, and no functional segregation of cell types [i.e., HTT, WRT, low-threshold thermoreceptive (LTT)]. This previous foundational study demonstrated how multisensory nociceptive neurons (i.e., WRT, HTT) responded separately to quantitative thermal and qualitative visual stimulation as well as established correlative relationships between the thermal stimulus intensity-response functions of neuronal discharge frequency and behavioral escape frequency. From these important findings about the role of area 7b in nociception and pain, we proposed (1, 2) that additional studies were needed to determine how multisensory nociceptive WRT-EN neurons integrate responses to both quantifiable somatic (thermal) and threatening visual stimuli and to determine the correlative relationship between the outcome of multisensory neuronal response integration and aversive escape behavior.

In ensuing years, human psychophysical studies, including some correlative results from blood oxygen level-dependent (BOLD)-functional MRI (fMRI) activity, have made a significant contribution to our understanding of how the cortex in general and multisensory nociceptive mechanisms in particular might integrate noxious somatosensory afferent input with threatening or salient visuosensory afferent input and influence behavioral outcome. Lloyd et al. (4) were first to report significantly increased BOLD-fMRI activity in the inferior parietal lobule (BA40) in response to threatening or potentially painful compared to nonthreatening or nonpainful mechanical stimuli presented within a visuospatial representation of surrounding peripersonal space related to the targeted somatic receptive field. They employed an embodied illusion of a real hand that was underneath a visually exposed and stimulated artificial hand. The results confirmed that multisensory nociceptive elements in BA40 were more activated by threatening visuosensory input in close visuotopic relationship to a “painful hand” than by nonthreatening visuosensory input related visuotopically to a “nonpainful hand.” Legrain and colleagues (5–14) showed that the spatial localization of nociceptive electrical stimulation of the hand can be modulated by varying the visuospatial alignment or distance of the salient visual object (e.g., light cue) in relation to the stimulated cutaneous receptive field. These observations highlight the importance of multisensory nociceptive neural mechanisms for integrating input from salient visual stimulation in peripersonal (near personal) space with input from noxious somatic stimulation in personal space and, consequently, for facilitating rapid threat detection and localization of noxious stimulation in two different but interrelated spaces and possibly for initiating immediate, guided action in defense of the body from potential injury. Their studies, however, have not addressed whether such multisensory nociceptive mechanisms modulate perceived pain magnitude in the same manner as they modulated pain localization. Also, further studies are needed to identify the specific locations of such multisensory nociceptive mechanisms. Additional studies by Iannetti and coworkers (15–19) using a paradigm for eyeblink induced by nonnociceptive electrical stimulation of the hand demonstrated the importance of defensive peripersonal (“egocentric”) space. The eyeblink reflex magnitude as measured by eye muscle electromyographic (EMG) activity was significantly enhanced when the stimulated hand entered the peripersonal space of the eyes and face and, seemingly paradoxically, regardless of whether the eyes were open or closed. The eyeblink reflex onset latency was shorter and its duration was longer when distances between hand and eyes were in near space compared to far space conditions. However, the specific multisensory sites for integrating threatening visuosensory, somatosensory (nonnociceptive and nociceptive), and possibly proprioceptive inputs to form such adaptable and defensive peripersonal spaces have not been clearly identified.

Based on the limited information about the somatosensory and visuosensory response properties of nociceptive neurons in area 7b from electrophysiological studies and on the paucity as well as ambiguity of extant information about nociception and pain in area 7b (BA40) from neuroimaging studies, the following questions need to be addressed to reaffirm and extend our knowledge about this sizable portion of the parietal association cortex (see Refs. 20–24). What role do multisensory nociceptive neurons in area 7b play in nociception, pain sensibility, and behavior, and what is the functional significance of their output to other interconnected cortical sites? Previous findings across several disciplines, including studies of clinical outcome from damage to area 7b (BA40) and adjacent areas (25–31), of extensive afferent and efferent cortical connectivity of area 7b (32–39), and of multisensory and sensorimotor (transformational) response properties of neurons in area 7b (1, 2, 40–49), provide a basis for the following general hypothesis: The subpopulation of multisensory nociceptive neurons in area 7b is a complex neural mechanism for *1*) integrating threatening visuosenory input with near noxious (43°C) and noxious (≥45°C) thermoreceptive input, for *2*) visuosensory enhancement of the near noxious (43°C) and mildly noxious (45°C) responses that is dependent on temporal alignment of multisensory inputs and on visual target spatial alignment with the thermoreceptive field (i.e., target direction, destination, and closing distance within peripersonal space), and for *3*) generating levels of sensorimotor transformational output (goal-directed coding) necessary to engage and regulate the appropriate intensity and duration of nocifensive behaviors (e.g., eye/head/body reorientation, defensive posturing and arm/hand movements) to minimize exposure and contact with the offending stimulus. Such sensorimotor output from area 7b would only contain information about a generalized location of the visuosensory and somatosensory stimuli because of the relatively crude somatotopic and “body-centered” visuotopic organization of neurons in area 7b (i.e., the entire or large portions of the head, arm, hand, or trunk). In the unisensory mode of this hypothetical neural mechanism, nocifensive behaviors are engaged when either a threatening visual target penetrates deep into peripersonal (near personal) space or an overtly noxious cutaneous stimulus is at or above pain threshold (≥47°C). The unisensory feature offers less injury protection because nocifensive behaviors are only engaged by overtly noxious stimulus intensities applied to skin at or above pain threshold or by a threatening visual target in close proximity to the skin receptive field. In the multisensory mode of operation, the appropriate temporal and spatial visuosensory target alignment would *1*) enhance the neuronal response to near noxious (43°C) and mildly noxious (45°C) cutaneous stimuli, *2*) in effect, temporarily “lower” the pain and nocifensive behavioral thresholds for avoidance or escape, and, consequently, *3*) avert impending or eventual tissue damage delivered by actual and significantly higher noxious stimulus intensities (≥47°C). The multisensory mode of the nociceptive neural mechanism in area 7b would serve as an early warning and defensive system that would initiate avoidance or escape behaviors before the possible delivery of strong noxious stimuli that would result in bodily injury. A multisensory, unlike a unisensory, mode of operation offers more injury protection because nocifensive behaviors are engaged preemptively at near noxious (43°C) and mildly noxious (45°C) thermal stimulus intensities below pain threshold (47°C) and such prepain stimulation is either contemporaneous with or preceded by threatening visual stimulation. The multisensory feature of prepain, early engagement of nocifensive behaviors may not be always advantageous and is prone to false positive warnings; the anticipated strong noxious stimuli and potential tissue injury may not occur. Repetition of this false positive warning could result in extinction of the multisensory evoked nocifensive behavior. However, it is conceivable that the top-down influence from other cognitive brain regions can make adjustments to minimize false positive warnings by altering the responsiveness of these area 7b nociceptive neurons in multisensory mode as well as the pain and nocifensive behavioral thresholds.

The aims of the present cellular study were *1*) to determine how multisensory nociceptive neurons (i.e., WRT-EN) in area 7b of the inferior parietal lobule integrate responses to threatening visuosensory input with near noxious (43°C) and noxious (≥45°C) somatosensory (thermoreceptive) input and *2*) to provide supportive evidence that neuronal processes in area 7b may underlie the multisensory integration of visuosensory and nociceptive somatosensory responses reported previously in human psychophysical studies. The spatial direction and proximity of visuosensory stimulation in peripersonal space in relation to somatosensory stimulation in personal space were emphasized in these studies of humans and in the present study of monkeys. The predicted electrophysiological and behavioral outcomes from the proposed hypothesis presented above were both tested under the same experimental conditions in a single monkey participant. Calibrated thermal and threatening visuosensory stimulation were applied, respectively, to the facial receptive field and spatially related peripersonal space of a highly trained monkey to perform an appetitive tolerance-escape task. The design of the study permitted a detailed examination of the temporal and spatial effects of threatening visuosensory input on the thermal stimulus-response (S-R) properties of multisensory nociceptive area 7b neurons and on the affiliated behavioral escape frequency and latency. The nocifensive behavioral metrics were determined by a hand-operated manipulandum used for volitional sensorimotor control of thermal onset and offset.

## METHODS

### Chronic Animal Preparation

The skull of the monkey (*Macaca mulatta*) was surgically implanted with two stainless steel receptacles held by titanium screws and acrylic cement for microelectrode positioning and recording devices and for a head restraint system. Surgery was performed under general anesthesia, aseptic conditions, and heart and respiratory monitoring in an accredited animal operating room and was followed by intensive postoperative care including monitoring vital signs and administration of analgesics and antibiotics (systemic and local around wound edges). The stainless steel internally threaded ring mount (2-cm diameter) for attaching a two-way sliding platform (*X*-*Y*-axis) and a hydraulic piston microdrive (*Z*-axis) during microelectrode recording was implanted on the skull overlying a craniotomy hole and the inferior parietal lobule (area 7b). An externally threaded sterile ring with a glued-on disk of Silastic sheeting (1 mm thick) was screwed into the ring mount to provide a watertight seal for protecting the underlying dura and reducing cortical pulsatile movement during microelectrode recording. A thin coating of antibacterial ophthalmic ointment to inhibit local infection was placed on the Silastic disk surface facing the dura. This entire assembly of rings was capped with a nylon plug when the monkey was in its home cage. Under aseptic conditions and general anesthesia, the Silastic ring insert in the ring mount was replaced weekly by another sterile one similarly coated with antibacterial ointment. At the same time, wound edges around the implanted skull receptacles were debrided, cleaned with disinfectant, and infiltrated with topical antibacterial ointment. The behavioral and electrophysiological recording procedures were approved by the University of Washington Animal Care Committee (animal welfare assurance no. A3464-01) and conformed to the guidelines issued by the National Institutes of Health for humane treatment of laboratory animals and to the guidelines for investigations of experimental pain in conscious animals issued by the Committee for Research and Ethical Issues of the International Association for the Study of Pain.

### Appetitive Tolerance-Escape Task

The monkey participant was studied electrophysiologically and behaviorally in a sound-attenuated chamber that was ventilated, illuminated or darkened, and monitored by video cameras. The monkey was habituated to a number of experimental conditions during each 3- to 4-h recording session. These included sitting quietly in a primate restraining chair, receiving fruit sauce or juice from a reinforcement delivery system, and restricting head movements by a restraining apparatus. No overt signs of discomfort were observed because the monkey was chair adapted, behaviorally shaped with food reinforcements, and attentively engaged in operant tasks to obtain food reward or escape noxious thermal stimulation. The monkey enclosure was also equipped with speakers and lights for delivery of auditory and visual cues and with a button switch to initiate and terminate experimental trials that included calibrated heating and cooling of skin and/or presentation of threatening visual stimulation near the skin on the face. A 3- to 4-h recording session was terminated prematurely if the monkey failed to initiate two or more consecutive trials following trial initiation prompts (light and auditory tone cues).

The appetitive tolerance-escape paradigm presented a conflict between two different reinforcements to the monkey, that is, a choice between a positive reward (acquiring fruit sauce or juice) and a negative reward (terminating noxious thermal stimuli). This behavioral model has desirable features for assessing behaviors evoked by thermal intensities above pain threshold (see Ref. 50). The paradigm ensures that *1*) the participant adopts conservative biases for aversive responding during the stimulus and operates within a stimulus intensity range that activates nociceptors when the pain tolerance threshold (50% escape responding) is met or exceeded; *2*) any avoidance component is eliminated by allowing the experimenter to determine the sequence of stimulus intensities; and *3*) escape frequency and latency (pain magnitude) in response to stimulus intensities can be measured from below pain tolerance threshold (<50% escape responding) to nearly 100% escape responding. During the appetitive tolerance-escape task (see also Fig. 1 of Ref. 1 for temporal sequence of stimulus and behavioral events), the monkey was reinforced with fruit sauce or juice for successfully performing the following sequence of behavioral responses: *1*) pressing an operant response button after the onset of a compound (yellow light and 1,900 Hz tone) discriminative stimulus (Sd1); *2*) pressing the response button throughout the presentation of a thermal stimulus applied to the face; and *3*) releasing the response button within 5 s of the onset of a second compound (red light and 2,900 Hz tone) discriminative stimulus (Sd2). Within the time interval between button press and onset of Sd2, thermal shifts were applied to skin on the maxillary face region using one of the following parameters: *1*) no thermal shift from a baseline adapting temperature of 38°C; *2*) cooling from an adapting temperature of 38°C to 32°C; or *3*) heating from an adapting temperature of 38°C to temperatures ranging from 41°C to 51°C. A 5-s duration was set for each steady-state or plateau temperature. The monkey was allowed to escape from the thermal stimulation at any time because a button release resulted in rapid cooling from temperatures > 41°C or rapid heating from a temperature of 32°C back to the baseline adapting temperature of 38°C. Food reinforcement was withheld on trials in which the monkey escaped thermal stimulation. For all thermal shifts, the interstimulus or intertrial interval (time between button release and the next Sd1 onset) was fixed at 25 s. A minimum delay time of 1.5 s was used between a button press and the onset of thermal shift and between the offset of thermal shift and onset of Sd2. These delays were intended to temporally differentiate any neuronal activity that may be motor task related to button press and/or release from any neuronal activity that may be sensory related to the onset and/or offset of a thermal shift. It should be noted that each thermal shift has dynamic heating and cooling components during rising and falling temperatures (10°C/s) relative to baseline and plateau temperatures. This may be especially relevant to thermal nociceptive neurons that have a wide dynamic response range and encode both innocuous and nocuous temperatures (i.e., WRT-EN). Blocks of three trials (each trial with the same thermal shift) were randomized so that blocks of high-temperature trials were alternated with blocks of low-temperature trials. Since the effects of motivation may affect pain tolerance and the perceived value of positive reinforcements during instrumental task performance, the daily ration of food and water before each experimental session was reduced but the daily feeding and watering schedule was held constant. Because the daily maintenance ration was not altered as a function of reward, the monkey was allowed to increase its body weight by increasing operant task performance. On this dietary regimen, the monkey maintained its baseline weight and the number of completed trials remained stable across recording sessions. Escape from an innocuous low temperature (i.e., 41°C) was observed only at the very end of each recording session and was intermixed with uninitiated trials when the monkey was prompted to button press by Sd1 cues (light and tone). Because this was an indication of food reward satiety and reduced motivation for performing the task, behavioral and electrophysiological results from any completed trials (3 or less) obtained during this latter portion of each session were not included in the data analysis. Electrophysiological data were also rejected from trials that were confounded by mechanoreceptive neuronal responses evoked by movements of the face against the thermal probe during thermal stimulation or during delivery of food reinforcement.

### Multisensory (Somatic and Visual) Stimulation and Electrophysiological Recording

Quantitative thermal stimulation of the skin on the maxillary region of the face was applied by a thermoelectric heat pump unit (Peltier type) operating under a servo-control system that included a thermocouple and cold junction compensator (electronic ice point reference circuit). The in-house manufactured and maneuverable thermal probe consisted of a heat pump assembly of two Peltier thermoelectric modules (p/n-type semiconductors) in a multistage stacked configuration that was cemented together by epoxy casting resin with high thermal conductivity. The four parallel ceramic plates in the stacked modules were arranged alternately as either a thermal source or a sink (hot or cold ceramic plate) depending on the same direction of current flow through both modules. The ceramic plate on one end of the heat pump assembly was attached to a copper thermal sink-water reservoir with inlet-outlet ports for attachment to a water circulating pump. A fine-wire copper-constantan thermocouple was cemented to the ceramic plate contact surface (12 × 15 mm) on the other end of the heat pump to measure the interface temperature between the heat pump contact plate surface and the skin. Skin temperature was raised or lowered at a rate of 10°C/s and held at predetermined set points and durations by program instructions from a microcomputer. Thermal shifts were applied at a constant rate of 10°C/s in all stimulation conditions throughout the study. The following categories of operational descriptors for “thermoreception” in the monkey participant were eventually formalized and based on the monkey’s escape frequencies and latencies elicited by a range of thermal shifts from a 38°C baseline to 41–51°C applied to a maxillary locus on the face: 41°C (nonnoxious); 43°C (nonnoxious or near noxious); 45°C (mildly noxious); 47°C (noxious, pain tolerance threshold, 50% behavioral escape responding); 49°C and 51°C (strongly noxious). Note that mildly noxious (45°C) and strongly noxious (≥49°C) stimuli, respectively, were designated to be below and above noxious (47°C) stimulation, which was the pain tolerance threshold (50% escape responding). These thermal descriptors were generally consistent with anecdotal observations reported by laboratory personnel when the same thermal temperatures were volitionally applied and terminated on a homologous maxillary locus of their face. The thermal descriptors relative to the pain tolerance threshold were also consistent with the escape frequency versus temperature functions observed in monkeys from our previous studies (1, 26). Moreover, linear regression coefficient (*r*^2^) analysis revealed that WRT-EN neurons graded noxious thermal stimulation intensity by increasing their mean discharge frequency in a monotonic manner to increasing noxious temperatures (≥45°C). The thermal S-R function of WRT-EN neurons closely approximated the stimulus intensity-escape frequency function as determined by correlation coefficient (*r*_s_) analysis [see Dong et al. (1)].

Qualitative mechanical stimulation of the face included brushing of hairs, innocuous pressure, and noxious pinch applied to the skin. The experimenter’s fingers were most often used to apply these familiar stimuli and were supplemented at times by the use of familiar air puffs from an ear syringe, camel hair brushes, forceps, and a blunt wooden probe. Quantitative threatening visual stimulation in peripersonal space was a needle (18 gauge) and a syringe (5 mL) attached to the tip of a maneuverable pneumatic piston device. The device was equipped with a strain gauge transducer and spring affixed to the piston to control and measure the distance of the piston with various air volume and flow rate delivered with a computer-controlled air pump. The syringe and needle could be maneuvered toward any horizontal and vertical point on the face, in any downward (elevation angle) or upward (depression angle) direction and horizon angle (azimuth) to a face locus, and in any closing angular distance to the face varying from 25 to 0 cm. The syringe and needle visual target approached and withdrew from the skin at a velocity of 25 cm/s. Displacements of the visual target were applied at a constant velocity of 25 cm/s in all stimulus conditions throughout the study. The needle tip never touched the skin at its closest point (0 cm), and a gap of ∼5 mm was maintained to mitigate contamination by possible mechanoreceptive responses, injury to the skin, and needle-evoked escapes unrelated to innocuous or nocuous thermal stimulation. The monkey never became accustomed to periodic injections of general and local anesthetics or antibiotics by displaying avoidance and aversive behaviors, and most likely an approaching needle and syringe and feint hypodermic injection to the face during experimental sessions were perceived also as threatening. It was just as likely that the strong association of an approaching needle and syringe with noxious thermal stimulation of the face and resultant escape during early training sessions was consequently perceived as threatening without a need to experience painful needle contact or penetration of facial skin. The effects of applying both threatening visuosensory and thermal somatosensory stimuli on multisensory neuronal activity and related behavioral escape frequency and latency were studied by two modes of stimulus presentation. In both modes described below, visual and thermal stimuli were spatially aligned by adjoining the visual target in peripersonal space to the thermally stimulated site in personal space, which was a skin locus on the contralateral maxilla overlying the infraorbital foramen. An approaching visual target was applied in a downward linear (“looming”) trajectory relative to the skin locus at an elevation angle of +45°, horizon angle of +45°, and velocity of 25 cm/s. In one mode of multisensory stimulation, visual and thermal stimuli (V + T) were spatially and temporally aligned such that the approaching visual target was held steady at various closing angular distances to the skin from a baseline 25-cm distance and for various durations (5 or 8 s) during which a thermal shift from a baseline temperature was held for 5 s at various steady (plateau) temperatures. The onset of visuosensory stimulation always preceded onset of thermal stimulation by 1.5 or 2.5 s to maintain a temporal order of visual to somatic events that is more common in nocifensive situations. After these events, both visual and thermal stimuli simultaneously returned to their respective baseline position and temperature. In the other mode of multisensory stimulation, visual and thermal stimuli (V → 2 s delay → T) were spatially aligned but temporally misaligned such that the approaching visual target was held steady at a 0-cm closing distance for 5 s, withdrew to the 25-cm baseline distance and then after a 2-s delay interval was followed by a thermal shift that was held for 5 s at various steady temperatures before returning to baseline temperature. The rationale for studying both temporal alignment and misalignment of threatening visuosensory and thermal somatosensory stimuli was based on a posteriori knowledge of how multisensory nociceptive neurons in cortical area 7b respond to each type of sensory stimuli in terms of their discharge frequency and adaptation characteristics (see Refs. 1–3) and a priori assumption of how these same evoked multisensory responses might be integrated when temporally coincident or separated and given the general principles of known types of multisensory integration observed in other cortical and subcortical sites in multiple species (see Refs. 51–53). The foveal position of the eyes relative to the threatening syringe-needle target was not monitored, and the monkey participant was not specifically trained for eye fixation or tracking of the target. It was assumed that the monkey had steadily and intently gazed at the approaching threatening target and steady target positions in peripersonal space based on the evoked visuosensory neuronal discharge.

Extracellular single-unit activity was recorded through a sterilized tungsten microelectrode that was insulated with borosilicate glass tightly bonded along most of its entire length except for the exposed tip (see Ref. 54). These in-house manufactured microelectrodes had tip diameters of <1 µm (15- to 25-µm exposed tip length) and impedances of 1.5–1.7 MΩ (1-kHz sine wave frequency, test current) and could withstand multiple penetrations of the dura mater and overlying Silastic disk without damage to their tip and insulation. Because of the microelectrode’s high flexural rigidity and streamlined penetrative shape, there was no need throughout the course of study to expose the dura during cell recording sessions and to periodically and aseptically remove any accumulation of thick reactive granular tissue overlying the dura. Titanium screws holding the implanted devices on the skull served as ground and reference electrodes. Unit activity was led to a high-impedance probe coupled to an AC preamplifier with a frequency response of 300 Hz to 3 kHz.

All isolated single units that have resting or spontaneous activity and a high signal-to-background noise ratio were tested for evoked activity by qualitative mechanical (blunt probe, forceps), thermal (nonpainful heated probe), and visual (novel objects, syringe and needle) stimulation to regions of the face. This initial and cursory qualitative examination with these handheld implements was performed to determine a cell’s sensory modality, receptive field location and size, and approximate response range for each modality. Unlike earlier studies that surveyed and characterized the functional cell types in cortical area 7b (1–3), this study was primarily focused on isolating and identifying a small population of multisensory nociceptive WRT-EN neurons with thermal and visual response properties related to the maxillary face region and on applying quantitative thermal and visual stimulation to study the interactive response properties under operant behavioral conditions. In each experimental session, the limited time available for electrophysiological recording (cell isolation and qualitative identification) in a restrained awake monkey and the limited number of trials for performing the appetitive tolerance-escape task before onset of satiety and lack of motivation to initiate trials had precluded a detailed quantitative study of most functionally identified neurons except for the prioritized multisensory nociceptive, WRT-EN neurons.

### Data Acquisition, Storage, Retrieval, and Analysis

During each recording session, the timelines and current status of task and stimulus parameters (i.e., sensory discriminative cues, time delays, thermal and visual stimuli) and behavioral responses (i.e., button press, release) were displayed graphically on a video monitor. Accumulated data of the task and stimulus parameters and accompanying behavioral responses (cued button press and release, noncued release/escape, and escape latency) from previous trials were displayed in tabular form and continuously updated after each trial. If necessary, the computer program allowed modification of task and stimulus parameters and their implementation in succeeding trials. Digital data from each trial that included task and stimulus parameters and behavioral responses and neuron identification number were coded and stored in computer data files. Analog signals of task and stimulus events and behavioral responses as well as extracellular activity were digitized and stored on compact discs for later analysis. Unit activity was screened online or off-line by using a dual time-amplitude window discriminator and displaying the discriminated spike activity along with the visual and/or thermal stimulation signals on a digital oscilloscope. A cumulative peristimulus time histogram (PSTH) of discharge frequency from three trials was initiated by sweep trigger pulses that preceded by 1.5 s the onset of either a thermal shift or an approaching visual target. This was useful to temporally discern background resting discharge activity and possible motor task activity (button press) from somatosensory or visuosensory evoked activity. The rationale for using a 100- or 200-ms bin width in peristimulus and poststimulus histograms was to readily resolve discharge adaptation characteristics at steady temperature plateaus or steady visual target closing distances and to resolve discharges to dynamic onset and offset of thermal shifts (10°C/s) or dynamic approach and withdrawal of a threatening visual target (25 cm/s). These bin parameters were used effectively in the previous study of multisensory, nociceptive neurons in area 7b (1). Alternative methods for discharge frequency analysis such as interspike interval or instantaneous spike frequency (1/time interval) were not used in this study because of the difficulty in counting the number of discharges in their natural temporal order over a stimulus time continuum [cf. Dong et al. (55)].

A modified total spikes method was used to determine evoked discharge frequency for each thermal shift with or without temporal overlap with a threatening visual stimulus [see Dong et al. (1)]. A cumulative poststimulus histogram of unit activity was generated from three trials of the same thermal shift and initiated by sweep trigger pulses at the start of the plateau temperature of 5-s duration. A tentative mean discharge frequency per second (MDF ± SE) was determined from the total number of discharges in 50 bins of 100-ms width over 5 s of plateau temperature from three consecutive trials. The final MDF (±SE) was determined by subtracting the tentative MDF from the MDF of 5 s of resting or spontaneous background discharges at the midpoint of the 25-s intertrial interval preceding the same three trials, which presumably avoided any discharge activity related to discriminative cues, motor tasks (i.e., button press, release), attention, or intention. The subtraction of resting or spontaneous background discharges from stimulus evoked discharges was intended to filter or average out the smaller but variable spontaneous background noise from possible contamination of the larger evoked signal. This was not a potential issue when studying evoked activity from trigeminal primary afferent fibers that did not have resting or spontaneous activity (55). From the preceding analysis, thermal stimulus-response (S-R) functions for each multisensory nociceptive neuron were constructed for three stimulus conditions: thermal stimulation only (T), temporally coincident visual and thermal stimulation (V + T), and temporally misaligned visual and thermal stimulation (V → 2 s delay → T). It should be noted that the MDF during the 5-s plateau temperature in all stimulus conditions was measured only from trials without escape because of the much lower number of escaped trials available at lower plateau temperatures and of the invalid statistical comparison of MDFs from variable durations (<5 s) at high plateau temperatures due to varying escape latencies. Both two-way ANOVA and Student–Newman–Keuls multiple comparison test (SigmaStat software) were performed on the S-R functions for the three stimulus conditions. Of particular importance was determining which MDFs in the V + T and V → 2 s delay → T conditions were significantly different (*P* < 0.05) from MDFs in the T condition. In a separate study of how visual target closing distances affect MDFs while maintaining noxious thermal stimulation constant, resultant MDFs were subjected to a one-way ANOVA and Student–Newman–Keuls multiple comparison test to determine which MDFs and related closing distances were significantly different (*P* < 0.05) from each other.

In previous studies of area 7b in conscious monkeys, an adequate sample size of nociceptive neurons was isolated among a large population of nonnociceptive neurons (1, 40). Only a relatively brief time period was necessary to record discharge activity from each nociceptive cell to determine their thermoreceptive and/or mechanoreceptive stimulus-response properties. The experimental goals of the present study required a formidable plan to electrophysiologically isolate and record activity at length from each cell in a population of only trigeminal, multisensory-nociceptive WRT-EN neurons in a monkey that performed an ethically accepted appetitive tolerance-escape task. The small number of WRT-EN neurons reported in the present study as in the previous study (1) and the incomplete acquisition of large stimulus-response datasets for some neurons precluded valid statistical analysis for the population of WRT-EN neurons. For example, to study the effects of temporal alignment and misalignment of multisensory responses (see Fig. 3 and Fig. 4) from an isolated WRT-EN neuron, 63 required and completed trials and ∼177 escaped trials were monitored over a minimum recording time of 2.5 h. Recordings from some WRT-EN cells were abruptly interrupted by losing microelectrode signal contact with the cell; for other WRT-EN isolated cells, the recording session was prematurely terminated because the monkey participant refused to initiate trials by prompting cues because of reduced motivation and satiety from food reward. Despite the small sample size of WRT-EN cells and constraints of available test trials in each experimental session, sufficient sample size and power for an individual cell permitted statistical determination of significant difference (*P* < 0.05) between stimulus testing conditions (T, V + T, V → 2 s delay → T) and their related independent variables (T at 41, 43, 45, 47, and 49°C). The same methodological conundrums were encountered when studying the effect of threatening visual target distances on responses evoked by noxious thermal stimulation (see results, Fig. 5). A false positive risk to consider is that statistical analysis of an adequate population of WRT-EN neurons may indicate no significant differences for some of these test conditions and related thermal or visual stimulation variables and, consequently, only weak inferences can be made about their general trends.

Monkey escape frequency and latency (pain magnitude) were measured to determine the behavioral S-R functions for two stimulus conditions: thermal stimulation only (T) and temporally coincident visual and thermal stimulation (V + T). Each data point in the S-R function represents the mean % escape frequency (±SE) of behavioral trials for the T or V + T stimulation condition from prior neuronal recording sessions and from eight sessions designated only for behavioral observations. A completed trial required no escape from a 5-s exposure to a temperature plateau (ranging from 41°C to 51°C) (T) or from a 5-s exposure to both temperature plateau (ranging from 41°C to 51°C) and threatening visual target (syringe and needle) held steady at a closing distance of 0 cm and 5-mm space gap from the skin (V + T). The pain tolerance threshold when using the appetitive tolerance-escape paradigm and applying only T stimulation to obtain an S-R function was assigned to a temperature that elicited 50% escape responding. Stimulation by only a threatening visual target (V) never elicited an escape response. Two-way ANOVA and Student–Newman–Keuls multiple comparison test were performed on the S-R functions for the two stimulus conditions (T and V+ T). Of particular interest was determining which mean escape frequencies in the V + T condition were significantly different (*P* < 0.05) from mean escape frequencies in the T condition. Mean escape latency (seconds) was extracted from the mean % escape frequency data. Escape latency was measured from the onset of a 5-s temperature plateau (ranging from 41°C to 51°C) (T) or from the onset of a 5-s exposure to both temperature plateau (ranging from 41°C to 51°C) and threatening visual (syringe and needle) held steady at a closing distance of 0 cm and 5-mm space gap from the skin (V + T). Use of statistical analysis to establish a significant difference for mean escape latencies between the V + T and T stimulation conditions, particularly at ≤43°C, was not possible because T stimulation alone at low temperatures elicited either few or no behavioral escapes.

## RESULTS

### Multisensory (Somatosensory and Visuosensory) Stimulation and Responses

A total of four wide-range thermoreceptive (WRT-EN) neurons in area 7b of the inferior parietal lobule were isolated by extracellular single-unit recording with stable high signal-to-noise ratios during lengthy test periods. These neurons were determined by quantitative visual and thermal stimulation to have visuosensory responses to an approaching and threatening object (syringe and needle) directed toward the face and somatosensory responses to thermal and mechanical stimulation of facial skin. Multisensory neurons were differentially responsive to independent variables in several methods of applying a threatening visual target, and these include *1*) a single direction for a target projecting to different skin loci (e.g., contralateral vs. ipsilateral); *2*) different directions (e.g., downward vs. upward) for a target projecting to the same skin locus; and *3*) different closing distances (e.g., far vs. near peripersonal space) between the threatening visual target and skin locus. The multisensory neurons were also differentially responsive to innocuous and nocuous thermal stimulation of facial skin covering a large bilateral receptive field but were only differentially responsive to innocuous mechanical stimulation (e.g., hair brushing, light touch and pressure) within the same cutaneous thermal receptive field.

**Figure 1.**
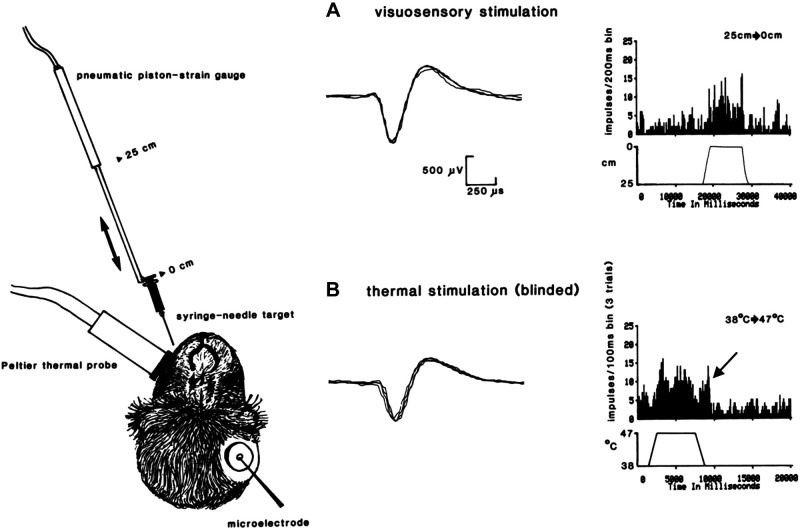
Somatosensory and visuosensory (multisensory) stimulation and responses. A wide-range thermoreceptive (WRT-EN) neuron that encoded differentially to nonnoxious and noxious thermal stimuli was studied in cortical area 7b. Depicted on *left* is a Peltier thermal probe in contact with skin on the contralateral maxillary region of the face and the visuosensory target (syringe and needle). The threatening visual target was presented by a pneumatic piston device along a downward linear trajectory (+45° elevation angle, +45° horizon or azimuth angle) in spatial alignment with the cutaneous receptive field at closing angular distances from 25 cm to 0 cm. The visual target never touched the skin at its closest point (0 cm), and a gap of ∼5 mm was maintained to mitigate contamination by possible mechanoreceptive responses. The visual target approached and withdrew from the skin at a velocity of 25 cm/s. The Peltier thermal stimulator delivered temperatures to the skin at a rate of 10°C*/*s. *A*: visuosensory response. The morphology of the extracellularly recorded spikes during visual stimulation is displayed in 4 overlapping traces. The peristimulus time histogram (PSTH) shows the responses evoked from 1 trial when the visual target was moving toward, held steady at a closing distance of 0 cm without skin contact, and withdrawn from the skin receptive field. *B*: thermoreceptive response. Spikes evoked during thermal stimulation have the same morphology as those evoked during visual stimulation. Thermal stimulation was delivered to the monkey in a darkened enclosure to eliminate contamination by visual responses (“blinded”). The PSTH depicts the responses evoked by thermal shifts from a baseline adapting temperature of 38°C to a noxious temperature of 47°C and cumulative activity from presentation of 3 trials with the same thermal shift. Note the afterdischarge (indicated by ←) during the offset of the thermal stimulus from 47°C. In all trials shown here, the monkey did not escape either the threatening visual or noxious thermal stimulation during performance of the appetitive tolerance-escape task (see rationale in methods).

Figure 1 depicts the location of the thermal probe on the skin of the contralateral maxillary region of the face and the pneumatic piston device used to project a threatening visual stimulus (syringe and needle) onto the same skin locus. The approaching visual object in peripersonal space is in spatial alignment or congruence with the thermal probe in personal space. Figure 1, *A* and *B*, display overlapping traces of action potentials from visuosensory and noxious thermal stimulation. The neuronal discharges of each sensory modality were identical in morphology (shape and size), recorded hours apart and without discharges from other neurons in the background, which strongly indicates that the visuosensory and thermosensory discharges are from only one neuron that is multisensory and nociceptive. In Fig. 1*A*, a peristimulus time histogram (PSTH) shows the visuosensory response evoked from a single trial by a threatening visual target delivered in a downward (looming) linear trajectory toward the skin at a +45° elevation angle, +45° horizon angle, velocity of 10 cm/s, and closing angular distance from 25 cm baseline to 0 cm end point (5-mm space between needle tip and skin), held close to skin for 8 s and withdrawn from the skin to the original 25 cm position at 10 cm/s. The threatening visual target was considered to be looming because the needle tip located at the 25 cm baseline position and directed at a +45° elevation angle and +45° horizon angle toward the skin locus was hovering at least 22 and 20 cm above the eyes and top of the head, respectively. Because the monkey’s head was restrained, an upward and lateral gaze was presumably necessary to clearly see the location and motion of the threatening target. However, presentation of threatening visual stimulation alone while using the above parameters and appetitive tolerance-escape task never elicited escape behavior. Note that no discharges were evoked beyond resting background activity by visual target retraction or maintaining the visual target at a steady 25-cm distance from the skin. The mean discharge frequency (minus resting background MDF) in response to threatening visual stimulation was ∼25 impulses/s. In Fig. 1*B*, thermal stimulation was delivered to the monkey in a darkened enclosure to eliminate possible contamination by visual responses (“blinded”). A PSTH depicts the cumulative responses evoked by three trials of identical thermal shifts delivered at a rate of 10°C/s from a baseline adapting temperature of 38°C to a noxious temperature of 47°C. The 47°C plateau temperature was held steady for 5 s before returning to baseline temperature at the same rate. The pain tolerance threshold (50% escape responding) of this monkey when using the appetitive tolerance-escape paradigm and applying only thermal stimulation to obtain an S-R function (5-s plateau temperature vs. escape frequency) was determined to be at 47°C. In this example, the monkey did not escape either the threatening visual stimulus or the noxious thermal stimulus trials during performance of the behavioral paradigm. Note the afterdischarge (indicated by ←) during the offset of the thermal stimulus from 47°C. Since this was a WRT-EN neuron that also responded to innocuous thermal temperature, residual heat within the skin during and after thermal stimulus offset may be one of the factors for the afterdischarges. Despite active cooling (10°C/s) from the Peltier thermal probe during temperature offset, there is a small discrepancy between the interface temperature measured between the heat pump contact surface and the skin and the actual intradermal temperature, which cannot be directly measured during an experimental session without damaging cutaneous receptors or affecting operant task behavior. The MDF (minus resting background MDF) in response to noxious thermal stimulation at the pain tolerance threshold was ∼27 impulses/s. In this unit, there was near parity of the MDF in response to threatening visuosensory stimulation compared to the MDF in response to a noxious thermal stimulation at 47°C (monkey pain tolerance threshold). It is evident that despite the near parity of MDFs in response to either threatening visual stimulation or noxious stimulation at 47°C (monkey pain tolerance threshold), the disparity between lack of escape from a visual threat compared to 50% escapes from painful thermal stimulation at 47°C suggests that the negative valence or behavioral aversiveness for each type of unisensory stimulation is unequal, at least within the experimental confine of an appetitive tolerance-escape operant task, which has a strong and conflicting food reward or positive valence component for not escaping. All four isolated WRT-EN neurons responded to threatening visuosensory stimulation or noxious thermal stimulation (47°C) with the same pattern and magnitude of discharge frequency.

**Figure 2.**
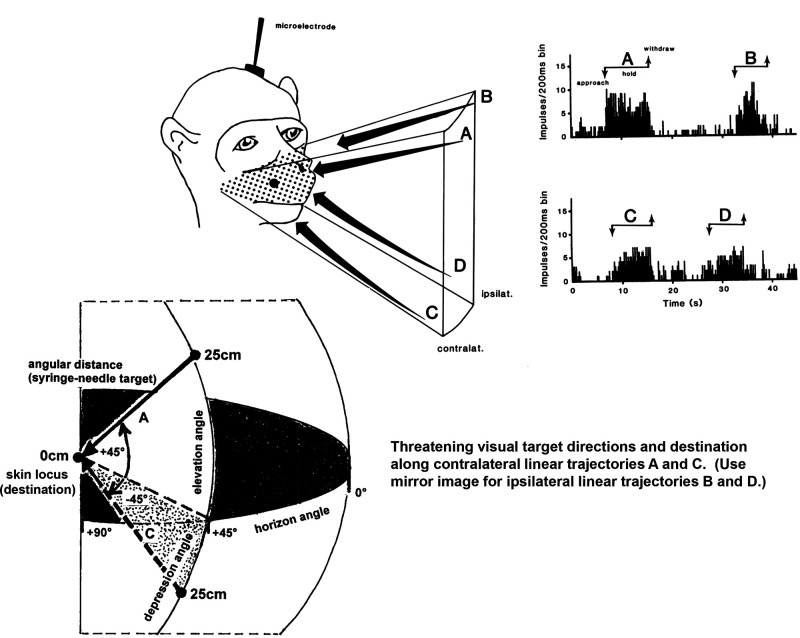
Effects of visual target trajectory direction and destination on multisensory neuron activity. The visuosensory responses were evoked from the same wide-range thermoreceptive (WRT-EN) neuron presented in Fig. 1. This neuron was also multimodal, that is, it responded to thermal and mechanical stimulation. The cutaneous receptive field was located bilaterally on the maxillary region of the face (stippled area). The cell responded differentially with a wide dynamic range to innocuous mechanical stimulation. A threatening visual target (syringe and needle) as depicted in the diagrams was presented along a downward (“looming”) linear trajectory (+45° elevation angle) to a maxillary locus in the contralateral (*A*) or ipsilateral (*B*) maxillary cutaneous receptive field and along an upward linear trajectory (−45° depression angle) to the same contralateral (*C*) or ipsilateral (*D*) maxillary locus. The horizon (azimuth) angle of the visual target was +45° or −45° relative to the contralateral or ipsilateral skin locus, respectively. The visual target traveled at a velocity of 10 cm/s and closing angular distance from 25 cm baseline to 0 cm end point (5-mm space between needle tip and skin), Observe in the peristimulus time histograms (PSTHs) the greater response magnitude (i.e., mean discharge frequency) of a threatening visual target applied in contralateral (*A*, *C*) vs. ipsilateral (*B*, *D*) trajectories and applied in downward (*A*, *B*) vs. upward (*C*, *D*) trajectories. The visuosensory response magnitude was dependent on both threatening target direction and destination in a wide visual receptive field that was spatially aligned with the bilateral somatic receptive field. Moreover, the somatosensory response magnitude to mechanical stimulation displayed a parallel dependency on destination, especially for contralateral vs. ipsilateral cutaneous loci (see also Ref. 1).

The directional and “destinational” effects of threatening visual target trajectories on a multisensory WRT-EN neuron are displayed in Fig. 2. The neuron was multimodal and responded to thermal or mechanical stimulation of a large skin receptive field located bilaterally on the maxillary region of the face. A threatening visual target (syringe and needle) was delivered to the face at a velocity of 10 cm/s and a closing angular distance from 25 to 0 cm (5-mm gap between needle tip and skin) to a locus on either the contralateral or ipsilateral maxilla and in either a downward (+45° elevation) or upward (−45° depression) linear trajectory. The horizon (azimuth) angle of the threatening visual target was +45° or −45° relative to the contralateral or ipsilateral skin locus, respectively. The PSTHs in Fig. 2 show the responses during the sequential application of an approaching target from 25 cm, holding target steady at a closing distance of 0 cm for 6–8 s, and withdrawing the target back to the 25 cm baseline position. No responses were seen beyond resting background activity during target retraction or presence of a stationary but visible target at a baseline distance of 25 cm. Note the greater response magnitude (i.e., MDF) of a threatening visual applied in contralateral (*A*, *C*) compared to ipsilateral (*B*, *D*) trajectories and applied in downward looming (*A*, *B*) compared to upward (*C*, *D*) trajectories. The visuosensory response magnitude was dependent on both direction and destination of a threatening visual target within a large bilateral visual receptive field that was spatially aligned with an equally large bilateral somatic receptive field. No escape behavior was observed in response to threatening visual stimulation applied in any direction or destination. Three WRT-EN neurons were tested for visuosensory responses to different directions and destinations of the threatening visual target, and all displayed the same differential discharge patterns and magnitudes. Likewise, the somatosensory response magnitude to innocuous mechanical stimulation displayed a parallel dependency on stimulus destination, namely, contralateral versus ipsilateral cutaneous loci (see also Ref. 1). The results strongly indicate that the optimal visuosensory response of multisensory WRT-EN neurons was evoked by an approaching threatening visual stimulus directed downward from a looming baseline position toward a contralateral cutaneous receptive field. In all the following studies described below, threatening visual stimuli were applied in a downward, contralateral trajectory toward the maxillary cutaneous receptive field at a fixed velocity of 25 cm/s and thermal shifts were applied directly to the same skin locus at a fixed rate of 10°C/s. These optimal stimulation parameters were fixed in subsequent studies to reduce the number of possible experimental variables.

### Effects of Temporal Alignment and Misalignment of Multisensory Stimulation and Responses

**Figure 3.**
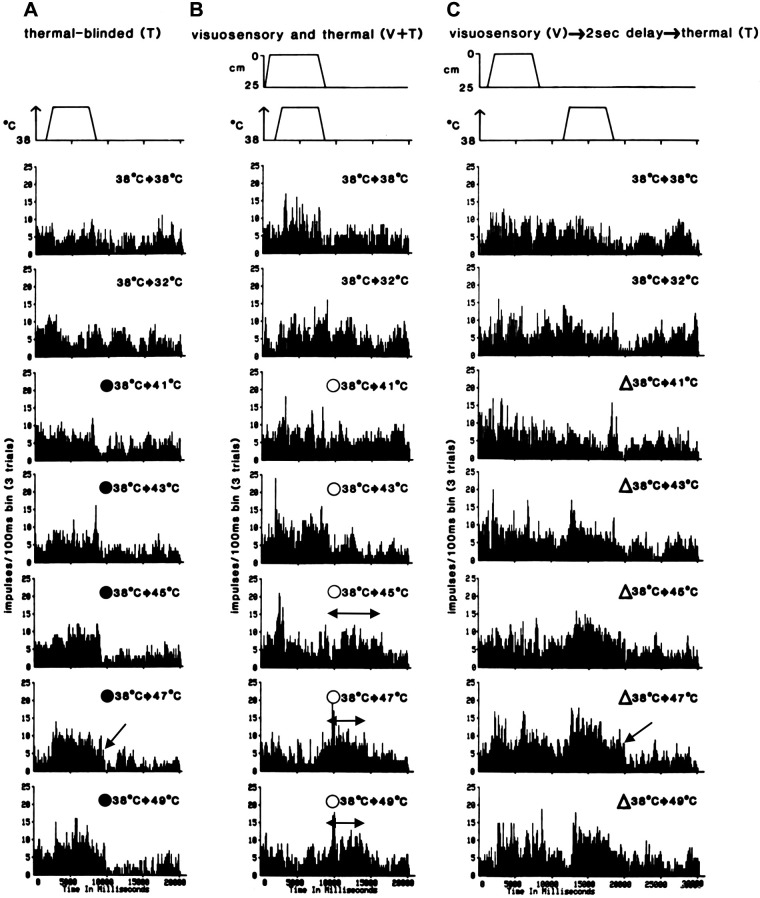
Interactions of visuosensory and somatosensory thermal responses. Visuosensory and/or thermal stimulation was applied by the apparatus described in Fig. 1 and Fig. 2 onto a spatially aligned visual and somatic locus within the contralateral maxillary face region. Threatening visuosensory stimulation (syringe and needle) was applied in a downward linear trajectory at 25 cm/s to a closing distance of 0 cm. No contact was made between needle and skin (5-mm gap). Evoked and background activity from a wide-range thermoreceptive (WRT-EN) neuron are displayed in peristimulus time histograms (PSTHs). Each PSTH represents cumulative activity from 3 trials with either thermal shifts from a baseline adapting temperature of 38°C (*A*) or thermal shifts from 38°C with temporally aligned or misaligned visual stimulation (*B* and *C*). All evoked responses were obtained while the monkey performed the appetitive tolerance-escape task to provide a means for escape from noxious thermal stimulation or for reward by tolerating noxious thermal stimulation. All data shown here were obtained from trials in which the monkey did not escape thermal, threatening visual, or multisensory stimulation (see rationale in methods). *A*: PSTHs of discharges evoked by thermals shifts above or below 38°C in the absence of illumination in the monkey enclosure (“blinded”). No discharges were evoked in the absence of thermal shifts (38°C → 38°C), and a weak but insignificant response to cooling (38°C → 32°C) was observed (shown in *top* 2 PSTHs). Increasing thermal shifts (●) from innocuous levels (38°C → 41–43°C) to noxious levels (38°C → 45–49°C) incrementally increased the mean discharge frequency (minus mean background discharge frequency) during the 5-s temperature plateau (see also Fig. 4). Note the afterdischarge (indicated by ←) during the offset of the thermal stimulus. *B*: temporal alignment of visually evoked and thermally evoked responses (V + T). Responses to a threatening visual stimulus (V) in the absence of a thermal shift (38°C → 38°C) or in the presence of a cooling thermal shift (38°C → 32°*C*) were observed but not significantly different from each other (shown in *top* 2 PSTHs). Complex response interactions were observed when a threatening visual stimulus (V) was temporally aligned with thermal shifts (○) from a baseline adapting temperature of 38°C to 41–49°C (T). Near noxious T shifts of 38°C to 43°C temporally aligned with V stimulation elicited a mean discharge frequency 100% greater than that from T shifts of 38°C to 43°C without V stimulation (compare PSTH in *A*). The mean discharge frequency of near noxious T + V was equivalent to the mean discharge frequency of noxious T shifts of 38°C to 46.5°C without V stimulation (see also Fig. 4). Unexpectedly, at noxious T shifts of 38°C to 45–49°C in temporal alignment with V, the mean discharge frequencies were significantly decreased below the mean discharge frequencies to T shifts of 38°C to 45–49°C without V stimulation (compare PSTHs in *A* and see also Fig. 4). Note that the depressed activity during V + T was followed by a prolonged poststimulus afterdischarge (indicated by ↔). The magnitude of the mean poststimulus discharge frequency was dependent on the magnitude of both the antecedent thermal shifts with V stimulation and the discharge depression. *C*: temporal misalignment of visually evoked and thermally evoked responses (V → 2 s delay → T). After a threatening visual stimulus (V) and 2-s delay, no thermal responses were observed in the absence of a thermal shift (38°C → 38°C) as expected or during a cooling thermal shift (38°C → 32°C) (see *top* 2 PSTHs). After antecedent V stimulation and 2-s delay, increasing thermal shifts (Δ) from innocuous levels (38°C → 41–43°C) to noxious levels (38°C → 45–49°C) increased incrementally the mean discharge frequency during the temperature plateau. After antecedent V stimulation and 2-s delay, near noxious T shifts of 38°C to 43°C elicited a mean discharge frequency 40% greater than that from T shifts of 38°C to 43°C without V stimulation (compare PSTH in *A*). The mean discharge frequency of near noxious T shifts following V stimulation and a 2-s delay was equivalent to the mean discharge frequency of noxious T shifts of 38°C to 45°C without V stimulation (see also Fig. 4). At noxious T shifts of 38°C to 45°C, the mean discharge frequency was 60% greater than that of noxious T shifts of 38°C to 45°C without V stimulation (compare PSTH in *A*). The mean discharge frequency of noxious T shifts of 38°C to 45°C was equivalent to the mean discharge frequency of noxious T shifts of 38°C to 47°C without V stimulation (see also Fig. 4). At noxious T shifts of 38°C to 47°C and 49°C, respectively, the mean discharge frequencies were slightly but insignificantly higher than those of noxious T shifts of 38°C to 47°C and 49°C without V stimulation (compare PSTHs in *A* and see also Fig. 4). Note the afterdischarge (indicated by ←) during the offset of the thermal stimulus.

**Figure 4.**
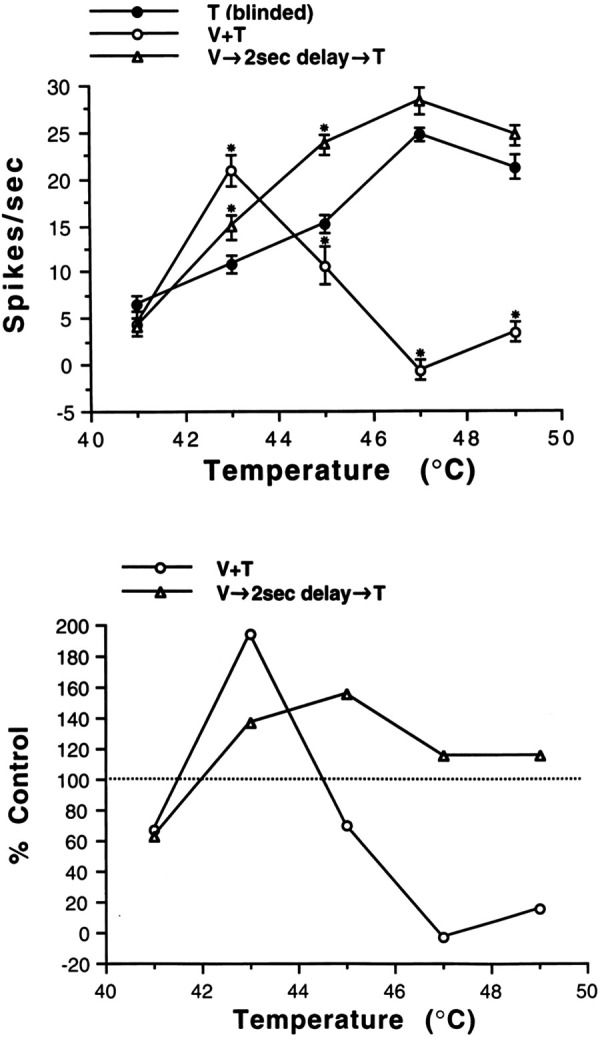
Effects of visuosensory stimulation on thermal stimulus-response (S-R) functions. The apparatus and methods for applying calibrated and controlled threatening visual stimulation (i.e., target destination, direction of linear trajectory, distance, and velocity) and thermal stimulation (i.e., temperature rise and fall rates, plateau duration) are described in Fig. 1 and Fig. 2. These graphs summarize the interaction of multisensory responses from a wide-range thermoreceptive (WRT-EN) neuron presented in Fig. 3. *Top*: thermal S-R functions plotted for experimental conditions of thermal stimulation only (T, ●), temporal alignment of visual and thermal stimulation (V + T, ○), and temporal misalignment of visual and thermal stimulation (V → 2 s delay → T, Δ). Each data point represents the mean discharge frequency ± SE (minus mean background discharge) during the 5-s plateau temperature of a thermal shift from 3 trials. It should be noted that in the V + T (○) condition the mean discharge frequency represents responses to both temporally aligned visual and thermal stimulation. A 2-way ANOVA and Student–Newman–Keuls multiple comparison test were performed on the S-R functions for the T vs. V + T and the T vs. V → 2 s delay → T conditions. *Mean discharge frequency that was significantly different (*P* < 0.05) from the mean discharge frequency elicited by only thermal stimulation. *Bottom*: responses from the V + T (○) and V → 2 s delay → T (Δ) experimental conditions are expressed as percentage of the responses from the T stimulation only condition, which is set at 100% (dotted line). Near noxious T shifts (38°C → 43°C) in the V + T and V → 2 s delay → T conditions, respectively, evoked mean discharge frequencies that were 100% and 40% greater than those from T stimulation only. The mean discharge frequencies of near noxious T shifts to 43°C for the V + T and V → 2 s delay → T conditions, respectively, were equivalent to the mean discharge frequencies of noxious T shifts to 46.5°C and 45°C without V stimulation (see *top*). Unexpectedly, application of noxious T shifts to 45°C in the V + T and V → 2 s delay → T conditions, respectively, evoked divergent mean discharge frequencies that were 30% less and 60% greater than those from T stimulation only. The mean discharge frequency of a noxious T shift to 45°C for the V → 2 s delay → T condition was equivalent to the mean discharge frequency of noxious T shifts to 47°C without V stimulation (see *top*). Note that mean discharge frequencies between the 2 stimulus conditions (V + T and V → 2 s delay → T) further diverged at noxious thermal shifts to 47°C and 49°C.

The temporal effects of threatening visuosensory input on the thermal stimulus-response properties of multisensory nociceptive neurons in area 7b are presented in Fig. 3 and Fig. 4. Innocuous and nocuous thermal stimulation (T, ●) were applied to the contralateral maxillary facial skin of a monkey during performance of the appetitive tolerance-escape task in a darkened enclosure (blinded). Application of a range of thermal shifts from 38°C to 41–49°C (10°C/s onset or offset rate, 5-s plateau temperatures, 3 trials/thermal shift) evoked activity displayed in Fig. 3*A* as cumulative PSTHs and plotted in Fig. 4 as an S-R function (5-s plateau temperatures vs. MDFs minus 5-s resting background MDFs). Increasing thermal shifts from innocuous levels (38°C to 41–43°C) to nocuous levels (38°C to 45–49°C) incrementally increased the MDF. Note that a cooling thermal shift from 38°C to 32°C evoked a weak but insignificant response. In Fig. 3, a short afterdischarge was often observed during the offset of innocuous and nocuous thermal stimulation (indicated by ←) in the T (●), V + T (○), and V → 2 s delay → T (Δ) conditions. Significant effects of threatening visuosensory input on the thermal S-R properties were observed (Fig. 3, *B* and *C*, and Fig. 4) when spatially aligned visuosensory and thermal stimulation were temporally aligned (V + T, ○) and misaligned (V → 2 s delay → T, Δ). Threatening visuosensory stimulation was applied in a downward (looming) linear trajectory from 25-cm distance to a closing distance of 0 cm on the contralateral maxillary facial skin (25 cm/s onset and offset rate, 5-s steady closing distance of 0 cm, 3 trials/closing distance). Responses to a threatening visual stimulus (V) in the absence of a thermal shift (38°C → 38°C) or in the presence of a cooling thermal shift (38°C → 32°C) were observed but not significantly different from each other. Of particular significance is the finding that at near noxious T shifts (38°C → 43°C) in the V + T and V → 2 s delay → T stimulation conditions, respectively, evoked MDFs were 100% and 40% greater than those from T stimulation only. The MDFs of near noxious T shifts to 43°C for the V + T and V → 2 s delay → T conditions, respectively, were equivalent to the MDFs of noxious T shifts to 46.5°C (approximately monkey pain tolerance threshold) and 45°C without V stimulation. The results suggest a linear summation of visual and thermal discharges from temporally aligned V + T (43°C) stimulation and a facilitation or augmentation of the thermal response by the antecedent visual response from temporally misaligned V → 2 s delay → T (43°C) stimulation. An a priori assumption based on unisensory visual or thermal discharge adaptation characteristics that the thermal response would be unaffected by the antecedent visual response and 2-s delay was incorrect. Despite the current lack of evidence from intracellular recordings in a WRT-EN multisensory neuron, the response outcome of V → 2 s delay → T stimulation may be due to antecedent visual input inducing prolonged and increased excitability [residual subthreshold or subliminal responses, excitatory postsynaptic potentials (EPSPs)] in the neuron beyond the visually evoked response discharge and, consequently, facilitated or augmented response discharge to subsequent thermal input.

**Figure 5.**
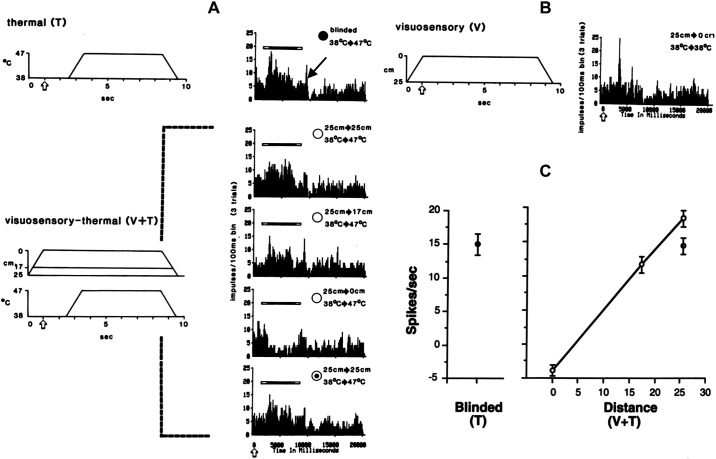
Effects of visual target distance on multisensory neuron activity. A wide-range thermoreceptive (WRT-EN) neuron in cortical area 7b that responded to a threatening visuosensory target (syringe and needle) and encoded innocuous and noxious thermal stimuli was studied for multisensory interactions. As shown in Fig. 3 and Fig. 4 (V + T condition), a combination of spatial and temporal alignment of a threatening visual target and near noxious temperature (43°C) significantly increased the mean discharge frequency relative to that evoked by near noxious temperature (43°C) alone. The mean discharge frequency was 100% higher and equivalent to that elicited by applying a 46.5°C noxious stimulus without visual stimulation. However, increasing thermal shifts to noxious levels at 45°C, 47°C, and 49°C gradually and significantly decreased the mean discharge frequency of the temporally aligned visual and thermal response. In this figure, a graded suppression of the temporally aligned multisensory response was also observed by decreasing the distance of the threatening visual target within the spatially aligned visual and somatic receptive fields (closing the target distance). Increasing the visual threat from peripersonal to near personal space elicited the same multisensory response depression as increasing the noxious thermal threat in personal space. All the evoked responses in this figure were obtained while the monkey performed the appetitive tolerance-escape task to provide a means for escape from noxious thermal stimulation or for reward by tolerating noxious thermal stimulation. All the data shown here were obtained from trials in which the monkey did not escape thermal, threatening visual, or multisensory stimulation. *A, top* peristimulus time histogram (PSTH): thermoreceptive response. Thermal stimulation (T, ●) was delivered to the contralateral maxillary region of the face in a darkened enclosure to eliminate contamination of by visual responses (“blinded”). The cumulative PSTH depicts the responses evoked by presentation of 3 trials with the same thermal shifts from a baseline adapting temperature of 38°C to a noxious temperature of 47°C at a rate of 10°C/s. The horizontal bar above the evoked activity indicates temperature rise or fall (open segment) and the final noxious 47°C plateau temperature (filled segment). The white arrow indicates the start of the PSTH. Note the afterdischarge (indicated by ←) during the offset of the thermal stimulus. *B*: visuosensory response. Threatening visual stimulation (V), syringe and needle, was presented in 3 trials along a downward linear trajectory from 25 cm to 0 cm and in spatial alignment with a cutaneous locus adjacent to the thermal probe tip. A gap of ∼5 mm between skin and needle tip was maintained to prevent possible contamination by mechanoreceptive responses. The threatening visual target approached and withdrew from the skin at a velocity of 25 cm/s. The cumulative PSTH depicts the responses evoked by presentation of 3 trials with the same visual stimulus parameters. The white arrows indicate the start of the PSTH and when the visual target reached 0 cm. *A, bottom* PSTHs: multisensory responses to spatially and temporally aligned V + T (○) stimulation. Threatening visual stimulation was presented at 3 closing distances: 25 cm to 25 cm (no change), 25 cm to 17 cm, or 25 cm to 0 cm. The white arrows indicate the start of each PSTH and when the visual target reached the designated closing distance to the skin. Each PSTH depicts the accumulated discharges of 3 trials for each closing distance. Observe the multisensory discharge frequency when the threatening visual target distance from the skin receptive field remained stationary at 25 cm and the increasing depression of the multisensory discharge frequency when the visual target moved toward the skin to steady distances of 17 cm and 0 cm. The discharge frequency depression was ameliorated by returning the visual target to the original stationary distance of 25 cm (⊙). *C*: graphs summarizing the results illustrated in *A* for noxious thermal stimulation alone (T, ●) and for spatially and temporally aligned V + T (○) stimulation at varying visual target, closing distances. A 1-way ANOVA determined a significant main effect for visual target distance, and a multiple comparison test (Student–Newman–Keuls) revealed that all the V + T (○) discharge frequencies evoked from various visual target closing distances were significantly different (*P* < 0.05) from each other. Note that a threatening visual target in peripersonal space at 25-cm distance from the skin was not effective in depressing the mean discharge frequency to noxious cutaneous stimulation at 47°C.

An unforeseen result emerged by applying mildly noxious T shifts from 38°C to 45°C in the V + T and V → 2 s delay → T stimulation conditions, respectively; this evoked divergent MDFs that were 30% less than and 60% greater than those from T stimulation only. The MDF of a noxious T shifts to 45°C for the V → 2 s delay → T condition was equivalent to the MDF of noxious T shifts to 47°C (monkey pain tolerance threshold) without V stimulation. The MDFs between the two stimulation conditions, V + T and V → 2 s delay → T, further diverged at greater noxious thermal shifts to 47°C and 49°C, as seen by additional MDF decrease in response to the V + T condition and by additional MDF increase in response to the V → 2 s delay → T condition. The incremental reduction of V + T activity from increasing T shifts at ≥45°C suggests an inhibitory process rather than absolute and relative refractoriness of summated visual and thermal discharges. The MDFs elicited at ≥45°C in the V → 2 s delay → T condition were significantly greater than the MDF elicited at 43°C in the V + T condition (see Fig. 4), and thus even at higher probabilities for discharge refractoriness in the V → 2 s delay → T condition, no inhibition (depression) of evoked responses was observed at any plateau temperature ≥ 45°C. Without intracellular recording of WRT-EN neurons, there remains some uncertainty about whether the reduction of V + T activity from increasing T shifts at ≥45°C as seen in Fig. 4 or from decreasing the closing distance of a visual threat as seen in Fig. 5 is an inhibitory [inhibitory postsynaptic potentials (IPSPs)] process. Two WRT-EN neurons were rigorously tested by completing the preceding large stimulus-response dataset for each neuron to determine the effects of temporal alignment and misalignment of multisensory stimulation and responses. Their resultant differential discharge patterns to different stimulus conditions (T, V + T, V → 2 s delay → T) and variable thermal shifts from 30°C to 41–49°C were similar.

Presented in Fig. 3*B* (cf. Fig. 5) is the only WRT-EN multisensory nociceptive neuron in this study in which the inhibited (depressed) activity during V + T (≥45°C) stimulation was followed immediately by a prolonged poststimulus afterdischarge (indicated by ↔). The increased magnitude of the poststimulus MDF covaried with the increased magnitude of both the antecedent thermal shifts with V stimulation and the discharge inhibition (depression). This prolonged poststimulus afterdischarge (↔) was unlike the brief afterdischarge (indicated by ←) during the offset of thermal stimulation. The prolonged poststimulus afterdischarge may be due to antecedent visual and somatic input inducing prolonged and increased excitability (residual subthreshold or subliminal responses, EPSPs) in the neuron beyond the reduced visual and somatic evoked response discharge and, consequently, facilitated or augmented discharge in response to the offset of thermal stimulation.

### Effects of Threatening Visual Target Distance on Noxious Thermoreceptive Responses (Near vs. Far Peripersonal Spaces)

The spatial effects of threatening visuosensory input on the thermal stimulus-response properties of multisensory nociceptive neurons in area 7b are presented in Fig. 5. The contribution of threatening visuosensory input, if any, to affect thermal responses was ineffective or diminished, respectively, when there was no approach of the visible threat toward the skin receptive field or the closing distance of the visible threat toward the skin receptive field did not reach 0 cm (near personal space). This is clearly observed in Fig. 5, *A* and *C*, during temporal coincidence of a stationary but visible threatening target at a baseline distance of 25 cm and a noxious thermal shift from 38°C to 47°C (V + T). The MDFs from noxious thermal stimulation alone (T, ●) compared to V + T [Fig. 5*A*, ○ (*top*) and ⊙ (*bottom*)] stimulation were not significantly different, which suggests that a stationary but visible threat at a distance of 25 cm added little or nothing to affect the noxious thermal discharge. Moreover, noxious thermal stimulation (47°C) and presentation of a stationary visual threat in far peripersonal space at a distance of 25 cm was insufficient to evoke V + T discharge inhibition or depression, which otherwise was seen for a target closing distance from 25 cm to 17 cm or to 0 cm [Fig. 5*A*, ○ (*middle*); see also Fig. 3*B* and Fig. 4]. As seen in all WRT-EN neurons, note the robust afterdischarge (indicated by ←) during the offset of the thermal stimulus from a noxious plateau temperature of 47°C.

The visuosensory response to a threatening looming visual target projecting downward along a contralateral linear trajectory, aligning spatially with the cutaneous receptive field and coming to rest at a closing distance from 25 cm to 0 cm, is shown in Fig. 5*B*. The visuosensory response to a target closing at an intermediate distance from 25 cm to 17 cm was disproportionately less (not shown). Observe the decremental effect that progressively decreasing the target closing distance from 25 cm to 25 cm (stationary), 25 cm to 17 cm, and 25 cm to 0 cm had on the MDF responding to thermal noxious shifts from 38°C to 47°C. In Fig. 5, *A* and *C* (○, V + T), MDFs were significantly reduced from 18 to 12 and finally to 4 discharges/s at each progressively shorter distance, respectively. The latter reduced MDF was even lower than the mean resting background discharge of 10/s and further suggests an inhibitory rather than absolute and relative refractory mechanism for evoked and resting discharge reduction (see also Fig. 4). Note the recovery of MDF when the visible threatening visuosensory was returned to the stationary and baseline 25 cm position (⊙, V + T). In comparison, increasing the visual threat to near personal space elicited the same multisensory response inhibition or depression as increasing the noxious thermal threat at ≥45°C in personal space (i.e., skin). Three WRT-EN neurons were tested for the effects of threatening visual target distances on noxious thermal responses, and all displayed the same differential discharge patterns and magnitudes. By way of contrast and reiteration of thermal discharge enhancement described above (see Fig. 3 and Fig. 4), threatening visuosensory stimulation delivered to a 0-cm closing distance (near personal space) also evoked discharges that summated with thermal discharges evoked by thermal shifts from 38°C to near noxious 43°C (V + T, temporal aligned condition) or evoked possible subthreshold or subliminal responses that facilitated or augmented thermal discharges evoked by a wide range of thermal shifts from 38°C to ≥43°C (V → 2 s delay → T, temporal misaligned condition).

### Effects of Unisensory (Thermal) and Multisensory (Visual and Thermal) Stimulation on Monkey Escape Frequency and Latency

**Figure 6.**
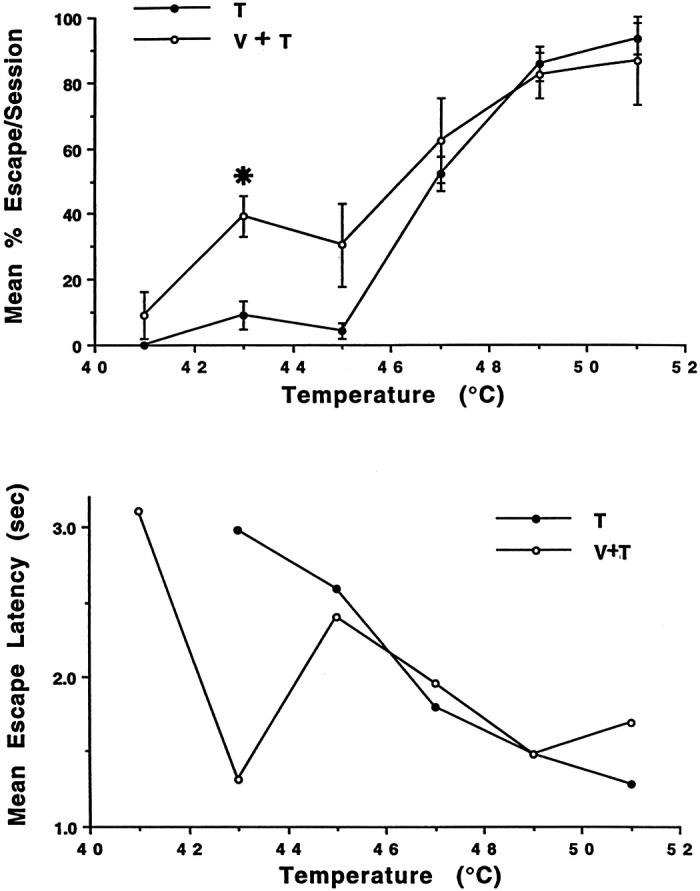
Effects of unisensory (thermal) and multisensory (visual and thermal) stimulation on monkey escape frequency and latency. All behavioral responses were obtained while the monkey performed the appetitive tolerance-escape task to provide a means for escape from noxious thermal stimulation or for reward by tolerating noxious thermal stimulation. The monkey volitionally initiated all trials after light and auditory cues. The appetitive tolerance-escape paradigm is described in detail in methods (see also Fig. 1 of Ref. 1). *Top*: behavior stimulus-response (S-R) functions plotted for experimental conditions of thermal stimulation only (T, ●) and temporal and spatial alignment of visual and thermal stimulation (V + T, ○). Each data point represents the mean % escape (±SE) of behavioral trials for the T or V + T stimulation condition from prior neuronal recording sessions and from 8 sessions designated only for behavioral observations. A completed trial required no escape from a 5-s exposure to a temperature plateau (ranging from 41°C to 51°C) (T, ●) or from a 5-s exposure to both temperature plateau (ranging from 41°C to 51°C) and threatening visual target (syringe and needle) held steady at a closing distance of 0 cm and 5-mm space gap from the skin (V + T, ○). Stimulation by only a threatening visual target (V) never elicited an escape response. A 2-way ANOVA and Student–Newman–Keuls multiple comparison test were performed on the behavioral S-R functions for the T vs. V + T conditions. *Mean % escape frequency elicited by V + T stimulation that was significantly different (*P* < 0.05) from the mean % escape frequency elicited by only thermal stimulation. The mean % escape frequency elicited by spatially and temporally aligned V + T stimulation at near noxious 43°C was significantly higher by 200% compared to the mean % escape frequency elicited by only T at near noxious 43°C. The mean % escape frequency elicited by V + T stimulation at near noxious 43°C was equivalent to the mean % escape frequency elicited by noxious T stimulation at 46.5°C without V stimulation. These % escape frequency results are consistent with the significant difference in wide-range thermoreceptive (WRT-EN) neuronal discharge frequencies between V + T (○) and T (●) conditions at near noxious temperature of 43°C (see Fig. 3*B* and Fig. 4). The pain tolerance threshold of this monkey when using the appetitive tolerance, behavioral escape paradigm and applying only T (●) stimulation was 47°C (50% escapes). The pain tolerance threshold (50% escape responding at 47°C) determined in the present study would be lower if the experimenter initiated the trials without the appetitive tolerance (positive reward) component and with only the escape (negative reward) component intact. Without an appetitive food reward, motivation to volitionally participate in thermal trials would be eliminated and pain thresholds would decrease (50% escape responding at temperatures lower than 47°C). *Bottom*: mean escape latency (seconds) derived from the mean % escape data. Escape latency was measured from the onset of a 5-s plateau temperature for each thermal shift (38°C → 41–51°C). The mean escape latency at a near noxious temperature of 43°C was dramatically decreased by multisensory V + T stimulation and was consistent with the significantly increased mean % escape frequency. Use of statistical analysis to establish a significant difference for mean escape latencies between the V + T and T stimulation conditions, particularly at ≤43°C, was not possible because T stimulation alone at low temperatures elicited either few or no behavioral escapes. The mean escape latency in response to V + T (43°C) stimulation appeared comparable to the mean escape latencies in response to delivering only overtly noxious T stimulation at 49°C and 51°C.

A psychophysical study was conducted to determine the relationship between escape frequency and latency in response to innocuous and nocuous thermal stimulation (T, ●) or in response to spatially and temporally aligned V + T stimulation (○). The electrophysiological S-R functions of a multisensory WRT-EN neuron (Fig. 4) established by using the same thermal (●) or threatening V + T stimulation (○) parameters were compared to their respective behavioral outcome. One note of caution in such comparisons is that the escape behavior also may be influenced by other unisensory and multisensory nociceptive neurons in cortical area 7b (i.e., HTT neurons) and elsewhere in the other nociceptive cortical areas [e.g., first somatosensory cortex (SI), SII, areas 7a and 5, insula, anterior and midcingulate cortex, etc.]. In Fig. 6, the mean % escape frequency (MEF) elicited by V + T stimulation at near noxious 43°C was significantly higher by 200% compared to the MEF elicited only by T stimulation at near noxious 43°C without V stimulation. Moreover, the MEF elicited by V + T stimulation at near noxious 43°C was equivalent to the MEF elicited only by T stimulation at noxious 46.5°C without V stimulation (approximately monkey pain tolerance threshold or 50% escapes). The enhancement of MEF at near noxious thermal stimulation (43°C) by the addition of threatening visuosensory stimulation in spatial and temporal alignment effectively lowered the pain tolerance threshold. Also, the mean escape latency in response to V + T at near noxious temperature of 43°C was reduced to a level comparable to mean escape latencies in response to delivering only noxious T stimulation at 49°C and 51°C. These behavioral findings are consistent with the significant increase of 100% between the MDFs in response to the same T(●) versus V + T (○) stimulation conditions (see Fig. 3*B* and Fig. 4). However, such enhancement of both MEF and MDF by V + T stimulation at near noxious temperature of 43°C was not seen when noxious temperatures were applied at ≥45°C. The MEF responses to V + T and T stimulation were not significantly different at noxious temperatures ≥ 45°C, which suggests that unisensory rather than multisensory thermal nociceptive neurons (i.e., WRT, HTT) may contribute solely to the escape responses at noxious temperatures ≥ 45°C. The MDFs of evoked and resting discharges from multisensory nociceptive WRT-EN neurons were strongly inhibited by V + T stimulation at noxious temperatures ≥ 45°C (see Fig. 3*B*, Fig. 4, and Fig. 5). The contribution from discharge inhibition (depression) seen in response to V + T stimulation at noxious temperatures of ≥45°C was not evident in the MEF, S-R function (Fig. 6). Spatially and temporally aligned V + T stimulation at temperatures ≥ 45°C incrementally reduced discharges and disengaged WRT-EN neurons from encoding noxious stimuli and possibly from contributing to escape behavior. WRT-EN neurons as part of a multisensory, early warning, defensive mechanism for body protection were engaged in a cascade of sequential events: *1*) linear summated discharges were evoked by threatening visual and near noxious thermal stimulation at 43°C to mimic equivalent discharges evoked solely by noxious thermal stimulation at 47°C, by which either discharge event could contribute to 50% escape responses (pain tolerance threshold), and *2*) if no escape occurred, discharge inhibition elicited by actual noxious thermal stimulation at ≥45°C would expeditiously reestablish or reset the resting baseline condition to resume detection of the next spatially and temporally aligned threatening visuosensory and near noxious thermal stimuli at 43°C; and *3*) the resulting enhanced evoked discharges could then again contribute to potential preemptive escape and avoidance of any forthcoming noxious thermal stimuli and pain. Emphasis should be given to the observation that the same WRT-EN neurons do have a different MDF, S-R function when presented with a temporally misaligned multisensory stimulation condition (V → 2 s delay → T; see Fig. 4), which might result in a different MEF, S-R function as well. Further studies are needed to determine the MDF and MEF, S-R functions of multisensory HTT-EN neurons with the same stimulus conditions (T only, V + T, and V → 2 s delay → T) that were used for WRT-EN neurons in order to establish a more complete assessment of multisensory integration in cortical area 7b.

## DISCUSSION

Multisensory nociceptive WRT-EN neurons in cortical area 7b of the inferior parietal lobule serve two important functions: *1*) in the unisensory mode of operation, these neurons either encode differentially with high fidelity the intensity of innocuous and nocuous thermal stimulation or encode differentially threatening visuosensory stimulation with independent variables such as direction, destination, or closing distance in visuospatial relation to the cutaneous receptive field, and *2*) in the multisensory mode of operation, these neurons encode both threatening visuosensory stimulation and the intensity of innocuous and nocuous thermal stimulation when both sensory modalities are in appropriate spatial and temporal alignment. The integrative process either by response summation (V + T) or response facilitation and/or augmentation (V → 2 s delay → T) significantly increased mean discharge frequencies above those evoked by thermal stimulation alone, particularly stimulation at near noxious (43°C) and mildly noxious (45°C) temperatures. In most cases, the enhanced multisensory evoked discharges were equivalent to the discharges evoked by noxious thermal stimulation alone at 47°C, which was also the monkey pain tolerance threshold (50% escapes). The monkey psychophysical study revealed that the mean escape frequency evoked by both threatening visuosensory and near noxious thermal (43°C) stimuli in spatial and temporal alignment (V + T) not only was significantly higher than that evoked by near noxious thermal stimulation alone at 43°C but was equal to that evoked by noxious thermal stimulation alone at ∼47°C, which was the monkey pain tolerance threshold (50% escapes). The importance of multisensory integration to nocifensive behavior was clearly demonstrated when only a combination of threatening visuosensory and near noxious thermal (43°C) stimulation in spatial and temporal alignment elicited high escape frequencies, whereas in unisensory mode threatening visuosensory stimulation alone elicited no escapes, and near noxious thermal (43°C) stimulation alone elicited only very few escapes. The remarkable concordance of elevating both neural discharge and escape frequency from a nonnociceptive and prepain level by near noxious thermal stimulation to a nociceptive and pain level by multisensory visual and near noxious thermal stimulation and integration is an elegantly designed defensive neural mechanism that in effect lowers both nociceptive response and pain thresholds to preemptively engage nocifensive behavior and, consequently, avert impending and actual injurious noxious thermal stimulation. The multisensory nociceptive and integrative mechanism in area 7b that enhances detection of weak but meaningful near noxious stimuli is undoubtedly a part of the larger multisensory defensive system in the “neural or pain matrix” that encodes and acts upon potentially damaging threats to the body as proposed by Iannetti and Mouraux (56), Legrain et al. (57), Morrison et al. (58), Mouraux and Iannetti (59), and de Vignemont (60).

Combined thermal (innocuous temperature, 43°C) and threatening visual stimulation that elicits both high multisensory neuronal discharge and escape frequency may be due partly to classical conditioning of the weaker innocuous thermal stimulus (CS) by the stronger threatening visual stimulus (US). Before stimulus conditioning, threatening visual stimulation (US) alone elicits both high neuronal discharge frequency and, presumably, high escape frequency (UR), whereas innocuous thermal stimulation (CS) alone elicits both low neuronal discharge frequency and low or negligible escape frequency (“NR”). If innocuous thermal stimulation (CS) elicits a “conditioned” response (CR) as a result of multisensory or paired visual threat and innocuous thermal stimulation (US + CS) over repeated trials, then eventually innocuous thermal stimulation (CS) alone would also evoke both higher neuronal discharge and escape frequencies (CR) like those responses (UR) evoked by US alone. Also, there is expectation after stimulus conditioning that a multisensory combination of threatening visual stimulation and innocuous thermal stimulation (US + CS) would evoke even greater neuronal discharge and escape frequencies (UR + CR) than those responses observed before stimulus conditioning (UR + NR). However, this was not what was observed in this study. Neuronal discharge frequency and escape frequency evoked by innocuous temperatures (**≤**43°C) or by threatening visuosensory stimulation alone remained the same throughout the study (see Fig. 4 and Fig. 6) and thus argue against any prior paired (US + CS) conditioning effect. Furthermore, applying thermal stimulation of ≤45°C alone while using the pain tolerance-escape behavioral paradigm elicited few or no escapes throughout the study (see Fig. 6). Despite the observation that threatening visual stimulation alone elicited a high discharge frequency throughout the study, no accompanying escapes were observed under the same behavioral paradigm throughout the study (see explanation of this result in the discussion below). Based on the few or absence of escape responses to either innocuous thermal or threatening visual stimulation, respectively, designation of these stimuli as “neutral, CS” or “potent, US” is inappropriate, Moreover, there is no suitable “neutral-potent” stimulus pair to form a conditioned response. Throughout the study, multisensory integration of a weak neuronal response to innocuous thermal 43°C stimulation and a strong neuronal response to threatening visual stimulation was an invariable linear summation of both responses, and such multisensory response integration was not influenced by stimulus conditioning. Also, significantly increased escape frequency was directly correlated to the significantly increased neuronal discharges by using the same multisensory stimulation (threatening visual and innocuous thermal 43°C stimuli).

### Single-Cell Electrophysiological Support of Human Multisensory Studies of Pain

Based on the electrophysiological results in this monkey study, cortical area 7b (BA40) of the inferior parietal lobule was confirmed as one of the sites for integrating multisensory responses evoked by threatening visuosensory and innocuous or nocuous somatosensory stimulation. This conclusion was fully or partly supported from results reported in human psychophysical and BOLD-fMRI studies using multisensory visual and somatic stimulation (4, 61). Numerous human and monkey studies using neuroimaging and electrophysiological methods have provided further supportive but often ambiguous evidence for a role of posterior parietal cortical area 7b (human homolog, Brodmann area 40) in nociception and pain perception. Studies have shown consistently that significant BOLD-fMRI or PET activity is differentially or exclusively elicited by noxious cutaneous stimulation and is located in the posterior parietal operculum, of which the second somatosensory cortex (SII) is a major occupant along with adjacent area 7b (PFop) and posterior insular cortex (granular and retroinsular) (62–89). Other lines of evidence (i.e., electrophysiological, neuroanatomical, clinical observations) also have provided support for the posterior parietal operculum, especially the SII or OP1 (90–92), as a crucial node in a distributed and interconnected cortical neural network (“matrix”) that also subserves pain among other nonpain sensory modalities (25–27, 40, 93–111). However, in most human studies to date, neuroimaged BOLD-fMRI and PET responses from the inferior parietal lobule (BA40), if any, were undifferentiated from nociceptive responses emanating from broad areas such as the entire posterior parietal cortex, “parasylvian” cortex, “operculoinsular” cortex, posterior parietal operculum, and intraparietal sulcus. Moreover, BOLD-fMRI and PET signals specifically related to the inferior parietal lobule (BA40 or area 7b), if any, were spatially and temporally conflated with signals from the adjacent SII, superior parietal lobule (BA7) or area 7a (PG), area 5 or even from the nearby posterior insula. Each of these cortical areas including area 7b is known to have its own unique set of thalamocortical and corticocortical connections as well as unique neuronal response properties dependent on a specific set of sensory, motor, sensorimotor, and attention-related inputs (1, 41, 65, 96, 107, 112–115).

Several conditions of visuosensory stimulation have been identified in both monkeys and humans that optimize multisensory nociception and pain perception. These optimal visuosensory stimulation conditions include *1*) identification of salient visual targets (threatening objects or cues); *2*) visual target spatial alignment or congruence with the somatic receptive field (downward looming direction and destinations on the same congruent side of the body) and close proximity of visual target to the somatic receptive field (near personal space); and *3*) movement of a salient visual target toward but not receding from the somatic receptive field (1, 2, 4–19, 116–122). Generally, the optimal multisensory responses in both monkeys and humans (i.e., neuronal discharge and escape frequency; speed of pain localization; magnitude, onset latency, and duration of defensive eyeblink reflex) were dependent on close proximity of the three-dimensional coordinates of the visual target in peripersonal space to those coordinates of the somatic stimulation site in personal space. Moreover, the visual receptive field was “body part centered,” such that moving the somatic receptive field in space moved the adjoining visual receptive field. Legrain and colleagues (5–14, 116–118) have demonstrated that the optimal detection speed or shortest detection latency for locating and responding to noxious cutaneous stimulation was observed by applying either a salient stationary cue light in close proximity to the cutaneous receptive field or salient moving lights within the peripersonal space that were approaching toward, in contrast to receding from, the associated cutaneous receptive field. Moreover, optimal localization of the stimulated hand receptive field was biased in favor of close proximity of the salient visual stimulus to the stimulated hand irrespective of its position in visual space (e.g., hand uncrossed vs. crossed, hand near vs. far), distance from the trunk, or incongruent gaze direction. Recent studies on sighted and early blind participants have shown that the integration of nociceptive somatotopic and “spatiotopic” maps into a common frame of reference is subject to plasticity and modulation depending on early visual and bodily experience and cognitive goals. Legrain and coworkers have demonstrated that optimal localization of nociceptive input from the hand when visual input from a defensive peripersonal space is both salient and dynamic depends on a multisensory integrative process for rapidly updating a common somatotopic (within body) and spatiotopic (outside body) coordinate system. Complementing these findings are those of Iannetti and colleagues (15–19) which suggested that the nonpainful but defensive eyeblink reflex induced by nonnociceptive electrical hand stimulation can be modulated by cognitive expectations, by barriers protecting the face from the hand, and by current and predicted stimulated hand positions with respect to the face. Recent studies of Iannetti and associates (119, 120) as well as others (121, 122) showed that the shape, dimension, and location of defensive peripersonal space and the magnitude of eye muscle EMG activity were dependent on additional factors including *1*) gravitational cues (e.g., looming threats above the head regardless of body position); *2*) approaching versus receding hand from the face; and *3*) distance and spatial alignment (congruence) of approaching objects that threaten the hand and/or face (e.g., using virtual reality arrows). The importance of spatial congruency in the visuotopic and somatotopic maps to pain and bodily defense was reported by Iannetti and colleagues (61, 123–125). They found that applying a noxious, painful (pinprick) radiant heat stimulation to the skin on one hand and crossing the hands (arms) over the body midline resulted in decreased magnitude of pain sensation and increased pain localization time compared to the uncrossed hand condition. This “multisensory analgesia” or “visually induced analgesia” has been attributed to seeing the stimulated hand and a mismatch between the somatotopic and body-centered visuotopic (spatiotopic) frames of reference. The defensive peripersonal space of the hand under their experimental conditions was presumably the entire congruent side of the body and not one in close proximity to the hand, irrespective of its uncross or crossed position [cf. DePaepe et al. (5, 6, 117, 118)]. Interestingly, Longo et al. (61) found increased BOLD-fMRI activity in the inferior parietal lobule (BA40) among other sites in the posterior parietal cortex and thus suggestive of a wide region for multisensory (visual and nociceptive) interaction during visually induced analgesia. However, the robustness of visually induced analgesia has been called into question based on the insignificant effect of direct vision of the stimulated hand on the perceived pain intensity (126).

Of particular significance was the observation that no monkey or human participants under highly controlled experimental conditions and highly trained to perform a specific operant task have been reported to escape or avoid the application of only threatening or salient visual stimulation. Threatening visual stimulation alone clearly activated area 7b neurons to nearly the same degree as overtly noxious thermal stimulation alone (i.e., 47°C), but it did not result in pain perception and escape. A possible explanation is that unisensory threatening visual stimulation and resultant evoked response are unable to engage sensorimotor nocifensive mechanisms that have become biased over time by cognitive influences to respond only to nociceptive thermal input or to multisensory threatening visual and innocuous or nocuous thermal inputs. These influential cognitive factors include heightened situational awareness, fear reduction, and prescience of task risk and reward, which all developed from familiarity of the controlled laboratory environment and prescribed repetitive task. Therefore, a threatening or salient visual stimulus if not accompanied by a detectable thermal stimulus decreases its probability of evoking pain, and thus becomes less dangerous, fearsome, and meaningful and unlikely to inflict tissue injury, and eventually reduces and eliminates motivation to escape (response extinction). In this same context, over time, a visual threat by itself has less negative stimulus valence. de Vignemont (60) has pointed out that nociceptive prediction and pain anticipation do not feel painful in humans. Threatening visuosensory stimulation that was likely to have similar predictive and anticipatory components when applied alone did not evoke monkey escapes. de Vignemont also proposed that nociceptive prediction and pain anticipation has an imperative bodily protection and defensive content when danger or impending tissue damage is imminent. Consistent with this proposed nocifensive role for nociceptive prediction and pain anticipation is the present experimental evidence that threatening visual stimulation when followed immediately by near noxious (43°C) and mildly noxious (45°C) thermal stimulation resulted in higher neuronal discharges and lower escape thresholds to preemptively avert impending and actual tissue damage.

### Threatening Visuosensory Stimulation and Visual Attention

Threatening visuosensory stimulation, such as an object approaching toward and proximate to the body, is a focus of robust stimulus-driven attention that contributes to avoidance of tissue injury and pain perception. This visual stimulus-driven attention may affect the responsiveness and selectivity of area 7b nociceptive neurons to specific types of visual threat. It is evident from the response properties of nociceptive neurons in area 7b (1) in monkey and from perceptual properties of acute pain in human (5, 7, 120) that selective and steady visual attention to familiar aversive objects and even unfamiliar nonaversive (novel) or familiar nonaversive objects depends on the object’s strength of association or disassociation with somatic nociception and pain. For example, familiar aversive objects such as a syringe and needle and familiar nonaversive objects such as cue light(s) in persistent association with somatic nociception and pain would both garner strong stimulus-driven attention. The source of complex visuosensory input to area 7b is primarily indirect from other areas of the posterior parietal cortex, namely area 7a (superior parietal lobule and adjacent intraparietal sulcus-LIP and VIP) and posterior parietal operculum (SII, insula) (see Refs. 32–39). Of particular importance is that area 7a directly receives significant complex visuosensory input from the medial superior temporal area (MST); in contrast, area 7b receives little, if any, direct input from MST (35, 38). MST and middle temporal area (MT) neurons may have a role in processing the approach and proximity of threatening visual stimuli and in selective visual attention because of their responses to linear local motion (velocity or acceleration), directional specificity, and depth perception (distance) of objects. MST neurons are engaged as well in “optic flow,” which is the perceived global motion of objects in the entire visual field expanding outward as a subject moves through the environment or “direction of heading” (see review in Ref. 36). Another source of complex visuosensory input to area 7b are direct and indirect (via area 7a) afferent inputs from the inferior temporal area (IT). Neurons in the IT have large visual receptive fields and may engage in cognitive activities such as visual learning, memory, and recognition of complex forms such as threatening or meaningful pain-related objects; these neurons are known to respond to visual recognition of a hand, face, facial expression, or critical features of such images (127).

Clinical studies have provided further insight into the relationship between threatening visuosensory stimulation and stimulus-driven, selective attention. Injury to the posterior parietal cortex, especially the inferior parietal lobule and posterior parietal operculum (area 7b, SII, and insula), alters the perception of pain (asymbolia for pain). The affective and motivational dimensions rather than the discriminative dimension of the pain experience were affected by the injury. For example, pain tolerance thresholds to noxious cutaneous stimulation were significantly higher or pain was absent in comparison to unaffected pain detection thresholds. A patient with pain asymbolia is either unaware of pain or aware of pain but unconcerned about it (loss of affect) and is not motivated to withdraw from the nociceptive stimulus. Pain asymbolia is often associated with the lack of nocifensive behaviors (escape or avoidance) in response to visual or verbal threats of bodily harm (1, 25–31). These clinical signs of pain asymbolia may be partly attributed to unilateral spatial neglect (inattention). This attentional deficit has been defined as a fundamental disturbance in the spatial distribution of selective visual and somatic attention that depends on multisensory integration, intracerebral representation of extrapersonal space, spatial orientation, and motivation-driven evaluation of behaviorally relevant sensory events (see Ref. 128). In patients with visual spatial hemi-neglect resulting from a forebrain stroke, the disordered spatial attention has a strong effect on the perception of both the location and quality of thermal pain stimuli (30).

Visual threat that has been strongly associated with somatic nociception and pain is inherently a primary focus of stimulus-driven attention. Visual threat itself does not evoke pain. This is based presently on the high discharge frequency elicited from multisensory WRT and HTT neurons in area 7b and the lack of behavioral escape in monkeys and pain reported in humans. The neuronal response to threatening or nonthreatening visual stimulus and, most likely, the strength of selective visuospatial attention are dependent on the complex form of an object and its static and dynamic properties in visual space. Threatening visual stimulation and selective visuospatial attention contribute to prepain prediction and anticipation and to pain-related discrimination, affect-motivation, and defensive behaviors. Similarly, thermal threat delivered at noxious intensities is a primary focus of stimulus-driven attention. Both thermal threat and selective somatotopic attention contribute to the discriminative, affective-motivational, and nocifensive dimensions of the pain experience. However, selective somatotopic attention may be more labile and much less robust when thermal stimulation is delivered at ≤45°C. This is based presently on low discharge frequency of WRT-EN neurons in area 7b and few, if any, escapes that occur during an operant task in monkeys and few reporting pain in humans. A well-designed human study (see Ref. 129) of thermal stimulus-driven attention has been performed using innocuous and noxious thermal stimulus intensities as well as using a directed and divided spatial attention paradigm. A similar experimental approach may determine how visual stimulus-driven attention is affected by threatening and nonthreatening objects that are in motion at different directions and destinations or are at different stationary distances in the near and far visual field.

### Timing of Multisensory Stimulation and Latency of Cortical Area 7b Neuronal Responses

This study was performed under the presumption that the most common temporal order of events in nocifensive situations is a visual peripersonal space event such as an approaching threatening or novel object followed by a somatic personal space event such as an innocuous or nocuous thermal or mechanical stimulus. This temporal order of events was maintained under both stimulus conditions used here, namely V + T and V → 2 s delay → T, and more so for the latter than the former stimulus condition. Despite the nearly complete temporal alignment of the V + T stimulation, the onset of threatening visual stimulation preceded by 1.5 or 2.5 s the onset of thermal stimulation (see Fig. 3*B* and Fig. 5). However, a precise measurement of multisensory response latencies and thus the onset of the integrative process was made difficult by possible temporal differences in the initial arrival of visual and trigeminal somatic inputs to the area 7b cortex. This dilemma was further exacerbated by an incomplete identification and electrophysiological understanding of the multiple and complex pathways that can carry visual and somatic inputs to multisensory nociceptive neurons in area 7b. For example, innocuous and nocuous thermal primary afferent input conducted slower over small myelinated Aδ and unmyelinated C trigeminal nerve fibers would alone contribute significantly to longer latencies and greater temporal dispersion before reaching area 7b than visual primary afferent input conducted faster over much larger myelinated Aβ optic nerve fibers. Without the use of very brief and superthreshold intensity for visuosensory (e.g., light flash) or somatosensory (e.g., laser pulse) stimulation to unnaturally synchronize excitation of primary afferent nerve fibers, a precise measurement of multisensory response latencies in area 7b neurons cannot be readily achieved. An earlier study reported that short-latency visuosensory discharges ranging from 50 to 290 ms (mean of 118 ms) in the adjacent area 7a (PG) of monkeys were evoked by LED light flashes (130). Whether or not visuosensory and thermal inputs evoke similar short-latency responses in area 7b (PF) is unknown but not unexpected when based on the approximate latencies of <1.5 s for neuronal discharges evoked by the onset of approaching and threatening visual or thermal stimulation in the present study. Short-latency thermal responses may be possible from evidence (see review in Ref. 1) that area 7b receives direct somatosensory input from many thalamic nuclei that contain nociceptive neurons (i.e., intralaminar, VPM, VPL, VPI, and pulvinar-lateral posterior nuclei) compared to less direct somatosensory input from cortical sites that contain nociceptive neurons (i.e., SI, SII, insula and cingulate cortex). Magnetoencephalography (MEG) recordings in human have shown that first and second pain elicited by laser pulse stimulation of presumably “Aδ” and “C” fibers in the hand evoked magnetic fields in the posterior parietal cortex with peak latencies of 183 ± 22 ms and 833 ± 22 ms, respectively (131). Another human MEG study reported that laser-evoked magnetic fields in response to hand stimulation and first pain (Aδ fibers) were precisely located in the inferior parietal lobule (BA40, area 7b) with a peak latency of 183 ± 7.2 ms (132). Manfron et al. (133, 134) have recently shown that to perceive simultaneously a visual stimulus (LED light flashes) with a thermonociceptive stimulus used to “selectively” activate either Aδ or C fiber afferent input (laser pulse) from the human hand, respectively, the thermal stimulus must precede the visual stimulus by an average of 76 ms or 577 ms. Nevertheless, the use of commonly and frequently encountered stimuli in the natural environment and the order, onset time, and relatively long duration in which they were applied in this study provided valuable insights into the integrative processes in area 7b. To study this integrative process at the cellular level, there was no need to artificially compensate for visual and thermal conduction differences by manipulating the order and onset time of visual and thermal stimulation to produce temporal simultaneity of multisensory inputs and responses. Optimal observations of multisensory visual and thermal input convergence and response integration in WRT-EN neurons were not compromised by the differences in peripheral and central conduction times between faster visual and slower somatic inputs. Fortuitously, in the V + T stimulation condition visuosensory input and response can be sustained by threatening visual stimulation applied in close proximity to the cutaneous receptive field for a long time period (e.g., >5 s). During this time period, slower-conducting cutaneous thermal input could steadily arrive by sustaining a temperature shift that allowed the resultant thermal response to temporally align and integrate with the visual response for at least 5 s (see Fig. 3*B*). Moreover, in the temporally misaligned visual and thermal stimulation condition (V → 2 s delay → T), there was no apparent temporal coincidence of visual and thermal discharge responses due to a 2-s stimulus delay interval, and yet residual antecedent V activity (possibly subthreshold) continued to affect the late-arriving thermal evoked input and related responses (see Fig. 3*C*).

### Putative Mechanism of Multisensory Nociceptive Integration in Area 7b Cortex

Evidence from temporally aligned (V + T) and misaligned (V → 2 s delay → T) threatening visual and thermal stimulation and inputs indicates that at least two multisensory integrative processes are involved in the visual enhancement of innocuous and nocuous thermal evoked discharges. Under the temporally aligned V + T stimulus condition, the stronger discharge frequency in response to threatening visual stimulation was linearly summed with the weaker discharge frequency in response to near noxious thermal stimulation at 43°C (see Fig. 4). The consequence of this linear summation was a total discharge frequency that was equivalent to the discharge frequency evoked by only a noxious thermal stimulus at 47°C, which was the monkey pain tolerance threshold (50% escapes). At both neuronal and behavioral levels, this discharge summation in effect lowered the threshold for nociceptive response, pain, and escape. A progressive inhibition or depression of discharges from stimulation by both threatening visual and thermal temperatures ≥ 45°C indicated that the gain of discharge frequency by discharge summation was preset and limited, and discharge inhibition or depression permitted the neuron to reset to its resting baseline condition (rearming the warning and defensive mechanism). Otherwise, an unlimited gain in multisensory discharge frequency by increasing thermal stimulation (≥45°C) would defeat the role of WRT-EN neurons as a warning and defensive mechanism to preemptively prevent actual noxious stimulation and tissue damage. Moreover, without a preset and limited gain control, a threatening visual stimulus in combination with a moderately noxious temperature could result hypothetically in very high discharge frequency and strong pain. The incremental inhibition (depression) of multisensory V + T activity in response to increasing temperatures ≥ 45°C was consistent with the “principle of inverse effectiveness” for multisensory integration in the superior colliculus of cats and monkeys (135, 136). In brief, as one weak unisensory stimulus (near noxious T, 43°C) becomes increasingly effective (V + T, 43°C), the magnitude of cell discharge enhancement to stimulus combinations (V +T, 45°C to 49°C) declines progressively (see Fig. 4). A low probability for engaging nocifensive behavior would need response enhancement by a strong threatening visual stimulus for detection of a weak or near noxious thermal stimulus, whereas a high probability for nocifensive engagement would not need response enhancement by a strong threatening visual stimulus for detection of an already strong or overtly noxious thermal stimulus.

Under the temporally misaligned (V → 2 s delay → T) condition for threatening visual and thermal stimulation and inputs, a different multisensory integrative process is involved in the visual enhancement of innocuous and nocuous thermal evoked discharges. Because of a 2-s delay between visual and thermal stimulation, no apparent coincidence of their respective discharges and thus no summation of visual and thermal evoked discharges was observed. Two seconds after cessation of threatening visual stimulation, near noxious (43°C) and mildly noxious (45°C) thermal stimulation evoked discharge frequencies, respectively, that were equivalent to discharge frequencies evoked by mildly noxious (45°C) and overtly noxious (47°C, monkey pain tolerance threshold) thermal stimulation alone. The “inverse effectiveness principle” can also apply to the V → 2 s delay → T stimulation condition. As one weak unisensory stimulus (near noxious T, 43°C or mildly noxious T, 45°C) becomes increasingly effective (V → 2 s delay → T, 43°C to 45°C), the magnitude of cell discharge enhancement to stimulus combinations (V → 2 s delay → T, 47°C to 49°C) declines progressively (see Fig. 4). The discharge enhancement from thermal and antecedent visual stimulation was nonlinear as well as preset with limits that did not significantly exceed the maximum discharge frequency ceiling of thermal stimulation alone at 47°C to 49°C. An integrative process that is likely to induce enhancement of weak thermal responses under this stimulus condition is prolonged facilitation or augmentation of thermal discharges by generation of excitatory subthreshold or subliminal activity (EPSPs) from antecedent strong and prolonged visual input. Intracellular recordings would be necessary to determine the frequency, strength, and time course of residual EPSPs generated by antecedent threatening visual input. A similar prolonged facilitation or augmentation of response discharge has been reported in other nociceptive neurons in the spinal cord dorsal horn and medial thalamus (137, 138). The phenomenon of discharge “windup” has been reported as a progressive increase in the discharge frequency and duration of a long-latency response burst to repetitive electrical stimulation of Aδ and C fibers at a fixed intensity and rate of 1 pulse/2 s. The duration of these enhanced and prolonged excitatory discharges far exceeded the estimated time for the slowest Aδ or C fiber input to arrive at the spinal cord or thalamus and was attributed to an incrementally prolonged facilitation or augmentation (subthreshold) process induced by the antecedent, iterative, and overtly noxious stimulation and input. The underlying molecular mechanisms for short-term synaptic plasticity such as facilitation, augmentation, and depression have not been fully elucidated in the mammalian central nervous system, especially in association cerebral cortices. However, in general, the decay time constant for facilitation has been reported with upper limits of hundreds of milliseconds, whereas the decay time constants for augmentation and depression have upper limits of seconds and even minutes (see Refs. 139–143).

### Sensorimotor Transformational Output of Area 7b Neurons for Controlling Nocifensive Behavior

In previous studies (1, 26), the thermal stimulus intensity-discharge frequency functions of WRT-EN and HTT-EN neurons in contrast to the S-R functions of WRT-NE and HTT-NE neurons closely approximated the thermal stimulus intensity-escape frequency functions. The unisensory thermal S-R functions of WRT-EN neuronal discharge frequency and escape frequency in the present study were also closely related and consistent with previous findings. More importantly, the significant enhancement of discharge frequency (linear summation or nonlinear facilitation/augmentation) by both near or mildly noxious thermal and near personal threatening visual stimulation was correlated to a significant increase in volitional escape frequency with short escape latency (premature release of a hand-operated response button during an appetitive tolerance-escape task). The multisensory nociceptive response in addition to its contribution to pain perception was potentially the earliest form of sensorimotor transformational output (goal-directed coding) for engaging and spatially guiding nocifensive behavior in peripersonal space. The cell discharge frequency under control by a preset and limited gain encoded the magnitude and time course of multisensory thermal and threatening visual stimulation. To achieve an adequate and nonadapting discharge frequency for an extended time period to initiate and sustain sensorimotor output and nocifensive behavior, the following requisite conditions for multisensory stimulation need to be fulfilled: *1*) visual and thermal stimuli should be spatially and temporally aligned or at least temporally applied in close succession; *2*) thermal stimulation should be sufficiently weak with respect to a limited response gain to accommodate visually evoked discharges; and *3*) strong visuosensory input should be maintained under optimal conditions by a threatening and approaching target and by spatiotopic congruency or close proximity of the target with respect to the thermal receptive field. Sensorimotor output should also contain spatial information about the locations of both incoming visual threat and somatic site threatened by injury. This is provided in part by the crude somatotopic and visuotopic organization of multisensory neurons in area 7b (PF), which represents predominantly the head/face/eyes and to a lesser extent the arm/hand/trunk (42–44, 48, 144). Localization of multisensory stimuli for engaging a nocifensive response is likely faster and less ambiguous by merging inputs from visual peripersonal (near personal) and somatic personal spaces that share the same common spatial coordinate system (or map) and integrative mechanism. Precise information about the location of multisensory visual and somatic stimuli from other cortical areas that are highly organized visuotopically and somatotopically (e.g., SI, SII, V1, V2) may contribute also to the sensorimotor stream.

Area 7b (PF) sends efferent fiber projections to many cortical areas that are known for multisensory, sensorimotor, and premotor integration. These include area 5, area PFG (between PF and PG), SII/PV, ventral premotor (PMv), anterior and ventral intraparietal (AIP, VIP), supplementary motor and somatosensory, and prefrontal, cingulate, and insular cortices (32, 33, 35–37, 115, 145–149). Such connectivity might permit somatomotor and visuomotor (oculomotor) neurons at some of these cortical sites to engage in complex nocifensive behavior and operate from motor coordinate systems that rely on the dynamic multisensory and spatial coordinate system provided in part by area 7b neurons. Andersen and colleagues (35, 47) have concluded from synthesis of voluminous experimental evidence that the posterior parietal cortex (PPC) generally performs sensory-to-motor transformations by converting between coordinate frames of spatial reference. In the transformation process, multisensory inputs are combined to create an abstract (or hybrid) and distributed representation of space or map of working space that may be used to guide movements. A distributed representation of eye-, head-, and body-centered spaces or their coordinates might be used by motor cortical sites to code appropriate defensive movements such as in nocifensive behaviors (see also Refs. 150–152). Area 7b in the inferior parietal lobule may be engaged in planning for movements (“intention”) among other cognitive functions (i.e., stimulus-driven attention and target selection) associated with the sensory-to-motor transformation process (see also Refs. 107, 112, 153, 154).

Recent studies by Morrison and coworkers (58, 155–157) have provided further insights into how nociceptive and painful information from area 7b (PF) and other PPC areas might influence motor-related regions of the cingulate and insular cortices and, consequently, volitional nocifensive behavior. Strong BOLD-fMRI activity has been reported (155) in the inferior parietal lobule (primarily area 7b or BA40) when human participants viewed pictures of other people’s hands grasping a threatening object (action recognition) that could be potentially noxious (pain recognition). This “mirroring” phenomenon may reflect the neuronal activity of the inferior parietal lobule in recognizing visual and somatic threats or pain as well as action of others and, perhaps, in generating sensorimotor transformational output (i.e., goal-directed coding or planning for movements) to reach out and to remove the threatening object from others (i.e., “empathetic action” that is imagined or real). Further studies with BOLD-fMRI (156, 157) revealed that the midcingulate cortex, especially the caudal cingulate motor zone, was robustly activated by painful noxious thermal stimulation when only linked with a volitional motoric response or action preparation and execution (button press) in contrast to other cortical areas such as the insula, which can be strongly activated by painful noxious thermal stimulation regardless of motoric response. Moreover, activation of the anterior cingulate (ACC) and midcingulate (MCC) cortices and insula was increased by the process of predicting whether a forthcoming thermal stimulus would be painful but was not modulated by the process of determining whether the current thermal stimulus was painful (sensory discrimination). Unlike the insula, modulation of AAC and MCC neural activity by pain prediction was dependent on whether a meaningful motoric response (button press) was performed to signal a predicted thermal stimulation that either reduced or did not reduce the duration of the forthcoming thermal stimulation. Furthermore, MCC activation was directly related with motor output. These results suggest that the role of ACC and MCC in pain processing was not tied to the sensory stimulus per se but was dependent on the effective motoric outcome of that stimulus for sensorimotor control of behavior. With respect to the strong reciprocal connectivity between BA40 (area 7b) and cingulate cortex (32, 33, 145, 146), neural processes in cingulate cortex underlying prediction of a painful event could potentially modulate nocifensive, sensorimotor activity in BA40 to defend against potential tissue damage and to minimize or avoid pain.

## DATA AVAILABILITY

Data will be made available upon reasonable request.

## GRANTS

This study was supported by grants from the National Institute of Neurological Disorders and Stroke (NS-29459) and the National Institute of Dental and Craniofacial Research (DE-07617).

## DISCLOSURES

No conflicts of interest, financial or otherwise, are declared by the author.

## AUTHOR CONTRIBUTIONS

W.K.D. conceived and designed research; performed experiments; analyzed data; interpreted results of experiments; prepared figures; drafted manuscript; edited and revised manuscript; and approved final version of manuscript.

## References

[1] Dong WK, Chudler EH, Sugiyama K, Roberts VJ, Hayashi T. Somatosensory, multisensory, and task-related neurons in cortical area 7b (PF) of unanesthetized monkeys. J Neurophysiol 72: 542–564, 1994. doi:10.1152/jn.1994.72.2.542. 7983518

[2] Dong WK, Chudler EH. Cortical nociceptive mechanisms. A review of neurophysiological and behavioral evidence in the primate. In: Forebrain Areas Involved in Pain Processing, edited by Besson JM, Guilbaud G, Ollat H. Paris: John Libby Eurotext, 1995, p. 183–195.

[3] Dong WK, Salonen LD, Kawakami Y, Shiwaku T, Kaukoranta EM, Martin RF. Nociceptive responses of trigeminal neurons in SII-7b cortex of awake monkeys. Brain Res 484: 314–324, 1989. doi:10.1016/0006-8993(89)90375-2. 2713690

[4] Lloyd D, Morrison I, Roberts N. Role for human posterior parietal cortex in visual processing of aversive objects in peripersonal space. J Neurophysiol 95: 205–214, 2006. doi:10.1152/jn.00614.2005. 16162829

[5] De Paepe AL, Crombez G, Legrain V. What’s coming near? The influence of dynamical visual stimuli on nociceptive processing. PLoS One 11: e0155864, 2016. doi:10.1371/journal.pone.0155864. 27224421 PMC4880339

[6] De Paepe AL, Crombez G, Legrain V. Remapping nociceptive stimuli into a peripersonal reference frame is spatially locked to the stimulated limb. Neuropsychologia 101: 121–131, 2017. doi:10.1016/j.neuropsychologia.2017.05.015. 28502633

[7] Filbrich L, Alamia A, Blandiaux S, Burns S, Legrain V. Shaping visual space perception through bodily sensations: testing the impact of nociceptive stimuli on visual perception in peripersonal space with temporal order judgments. PLoS One 12: e0182634, 2017. doi:10.1371/journal.pone.0182634. 28777824 PMC5544212

[8] Filbrich L, Alamia A, Burns S, Legrain V. Orienting attention in visual space by nociceptive stimuli: investigation with a temporal order judgment task based on the adaptive PSI method. Exp Brain Res 235: 2069–2079, 2017 [Erratum in Exp Brain Res 235: 2901, 2017]. doi:10.1007/s00221-017-4951-2. 28374087

[9] Filbrich L, Halicka M, Alamia A, Legrain V. Investigating spatial characteristics of the crossmodal interaction between nociception and vision using gaze direction. Conscious Cogn 57: 106–115, 2018. doi:10.1016/j.concog.2017.11.011. 29207312

[10] Filbrich L, Blandiaux S, Manfron L, Farnè A, De Keyser R, Legrain V. Unimodal and crossmodal extinction of nociceptive stimuli in healthy volunteers. Behav Brain Res 362: 114–121, 2019. doi:10.1016/j.bbr.2019.01.002. 30630019

[11] Vanderclausen C, Filbrich L, Alamia A, Legrain V. Investigating peri-limb interaction between nociception and vision using spatial depth. Neurosci Lett 654: 111–116, 2017. doi:10.1016/j.neulet.2017.05.060. 28578106

[12] Vanderclausen C, Manfron L, De Volder A, Legrain V. The influence of visual experience and cognitive goals on the spatial representations of nociceptive stimuli. Pain 161: 328–337, 2020. doi:10.1097/j.pain.0000000000001721. 31613868

[13] Vanderclausen C, Bourgois M, De Volder A, Legrain V. Testing the exteroceptive function of nociception: the role of visual experience in shaping the spatial representations of nociceptive inputs. Cortex 126: 26–38, 2020. doi:10.1016/j.cortex.2019.12.024. 32062141

[14] Manfron L, Legrain V, Filbrich L. Seeing or not seeing where your hands are. The influence of visual feedback about hand position on the interaction between nociceptive and visual stimuli. Multisens Res 33: 457–478, 2020. doi:10.1163/22134808-20191448. 31648189

[15] Sambo CF, Forster B, Williams SC, Iannetti GD. To blink or not to blink: fine cognitive tuning of the defensive peripersonal space. J Neurosci 32: 12921–12927, 2012. doi:10.1523/JNEUROSCI.0607-12.2012. 22973016 PMC6703813

[16] Sambo CF, Liang M, Cruccu G, Iannetti GD. Defensive peripersonal space: the blink reflex evoked by hand stimulation is increased when the hand is near the face. J Neurophysiol 107: 880–889, 2012. doi:10.1152/jn.00731.2011. 22090460

[17] Sambo CF, Iannetti GD. Better safe than sorry? The safety margin surrounding the body is increased by anxiety. J Neurosci 33: 14225–14230, 2013. doi:10.1523/JNEUROSCI.0706-13.2013. 23986256 PMC6618504

[18] Bufacchi RJ, Liang M, Griffin LD, Iannetti GD. A geometric model of defensive peripersonal space. J Neurophysiol 115: 218–225, 2016. doi:10.1152/jn.00691.2015. 26510762 PMC4760470

[19] Wallwork SB, Talbot K, Camfferman D, Moseley GL, Iannetti GD. The blink reflex magnitude is continuously adjusted according to both current and predicted stimulus position with respect to the face. Cortex 81: 168–175, 2016. doi:10.1016/j.cortex.2016.04.009. 27236372 PMC4962765

[20] Caspers S, Geyer S, Schleicher A, Mohlberg H, Amunts K, Zilles K. The human inferior parietal cortex: cytoarchitectonic parcellation and interindividual variability. Neuroimage 33: 430–448, 2006. doi:10.1016/j.neuroimage.2006.06.054. 16949304

[21] Gregoriou GG, Borra E, Matelli M, Luppino G. Architectonic organization of the inferior parietal convexity of the macaque monkey. J Comp Neurol 496: 422–451, 2006. doi:10.1002/cne.20933. 16566007

[22] Caspers S, Eickhoff SB, Rick T, von Kapri A, Kuhlen T, Huang R, Shah NJ, Zilles K. Probabilistic fibre tract analysis of cytoarchitectonically defined human inferior parietal lobule areas reveals similarities to macaques. Neuroimage 58: 362–380, 2011. doi:10.1016/j.neuroimage.2011.06.027. 21718787 PMC8007958

[23] Caspers S, Schleicher A, Bacha-Trams M, Palomero-Gallagher N, Amunts K, Zilles K. Organization of the human inferior parietal lobule based on receptor architectonics. Cereb Cortex 23: 615–628, 2013. doi:10.1093/cercor/bhs048. 22375016 PMC3563340

[24] Niu M, Rapan L, Funck T, Froudist-Walsh S, Zhao L, Zilles K, Palomero-Gallagher N. Organization of the macaque monkey inferior parietal lobule based on multimodal receptor architectonics. Neuroimage 231: 117843, 2021. doi:10.1016/j.neuroimage.2021.117843. 33577936 PMC8188735

[25] Greenspan JD, Winfield JA. Reversible pain and tactile deficits associated with a cerebral tumor compressing the posterior insula and parietal operculum. Pain 50: 29–39, 1992. doi:10.1016/0304-3959(92)90109-O. 1513603

[26] Dong WK, Hayashi T, Roberts VJ, Fusco BM, Chudler EH. Behavioral outcome of posterior parietal cortex injury in the monkey. Pain 64: 579–587, 1996. doi:10.1016/0304-3959(95)00215-4. 8783324

[27] Greenspan JD, Lee RR, Lenz FA. Pain sensitivity alterations as a function of lesion location in the parasylvian cortex. Pain 81: 273–282, 1999. doi:10.1016/S0304-3959(99)00021-4. 10431714

[28] Berthier M, Starkstein S, Leiguarda R. Asymbolia for pain: a sensory-limbic disconnection syndrome. Ann Neurol 24: 41–49, 1988. doi:10.1002/ana.410240109. 3415199

[29] Veldhuijzen DS, Greenspan JD, Kim JH, Lenz FA. Altered pain and thermal sensation in subjects with isolated parietal and insular cortical lesions. Eur J Pain 14: 535.e1–535.e11, 2010. doi:10.1016/j.ejpain.2009.10.002. 19939715 PMC2872197

[30] Liu CC, Veldhuijzen DS, Ohara S, Winberry J, Greenspan JD, Lenz FA. Spatial attention to thermal pain stimuli in subjects with visual spatial hemi-neglect: extinction, mislocalization and misidentification of stimulus modality. Pain 152: 498–506, 2011. doi:10.1016/j.pain.2010.10.017. 21111534 PMC3403741

[31] Griffith T, Kind A. Pain asymbolia is not pain. Philosophy of Science 2023: 1–18, 2023. doi:10.1017/psa.2023.167.

[32] Cavada C, Goldman-Rakic PS. Posterior parietal cortex in rhesus monkey: I. Parcellation of areas based on distinctive limbic and sensory corticocortical connections. J Comp Neurol 287: 393–421, 1989. doi:10.1002/cne.902870402. 2477405

[33] Cavada C, Goldman-Rakic PS. Posterior parietal cortex in rhesus monkey: II. Evidence for segregated corticocortical networks linking sensory and limbic areas with the frontal lobe. J Comp Neurol 287: 422–445, 1989. doi:10.1002/cne.902870403. 2477406

[34] Cavada C, Goldman-Rakic PS. Topographic segregation of corticostriatal projections from posterior parietal subdivisions in the macaque monkey. Neuroscience 42: 683–696, 1991. doi:10.1016/0306-4522(91)90037-O. 1720224

[35] Andersen RA, Asanuma C, Essick G, Siegel RM. Corticocortical connections of anatomically and physiologically defined subdivisions within the inferior parietal lobule. J Comp Neurol 296: 65–113, 1990. doi:10.1002/cne.902960106. 2358530

[36] Andersen RA, Snyder LH, Bradley DC, Xing J. Multimodal representation of space in the posterior parietal cortex and its use in planning movements. Annu Rev Neurosci 20: 303–330, 1997. doi:10.1146/annurev.neuro.20.1.303. 9056716

[37] Lewis JW, Van Essen DC. Corticocortical connections of visual, sensorimotor, and multimodal processing areas in the parietal lobe of the macaque monkey. J Comp Neurol 428: 112–137, 2000. doi:10.1002/1096-9861(20001204)428:1<112::aid-cne8>3.0.co;2-9. 11058227

[38] Rozzi S, Calzavara R, Belmalih A, Borra E, Gregoriou GG, Matelli M, Luppino G. Cortical connections of the inferior parietal cortical convexity of the macaque monkey. Cereb Cortex 16: 1389–1417, 2006. doi:10.1093/cercor/bhj076. 16306322

[39] Goldring AB, Cooke DF, Baldwin MK, Recanzone GH, Gordon AG, Pan T, Simon SI, Krubitzer L. Reversible deactivation of higher-order posterior parietal areas. II. Alterations in response properties of neurons in areas 1 and 2. J Neurophysiol 112: 2545–2560, 2014. doi:10.1152/jn.00141.2014. 25143537 PMC4233279

[40] Robinson CJ, Burton H. Somatic submodality distribution within the second somatosensory (SII), 7b, retroinsular, postauditory, and granular insular cortical areas of *M. fascicularis*. J Comp Neurol 192: 93–108, 1980. doi:10.1002/cne.901920106. 7410615

[41] Frot M, Faillenot I, Mauguière F. Processing of nociceptive input from posterior to anterior insula in humans. Hum Brain Mapp 35: 5486–5499, 2014. doi:10.1002/hbm.22565. 24916602 PMC6869247

[42] Leinonen L, Hyvärinen J, Nyman G, Linnankoski I. I. Functional properties of neurons in lateral part of associative area 7 in awake monkeys. Exp Brain Res 34: 299–320, 1979. doi:10.1007/BF00235675. 105918

[43] Leinonen L, Nyman G. II. Functional properties of cells in anterolateral part of area 7 associative face area of awake monkeys. Exp Brain Res 34: 321–333, 1979. doi:10.1007/BF00235676. 105919

[44] Hyvärinen J. Regional distribution of functions in parietal association area 7 of the monkey. Brain Res 206: 287–303, 1981. doi:10.1016/0006-8993(81)90533-3. 7214136

[45] Hyvärinen J. Posterior parietal lobe of the primate brain. Physiol Rev 62: 1060–1129, 1982. doi:10.1152/physrev.1982.62.3.1060. 6806834

[46] Mountcastle VB, Lynch JC, Georgopoulos A, Sakata H, Acuna C. Posterior parietal association cortex of the monkey: command functions for operations within extrapersonal space. J Neurophysiol 38: 871–908, 1975. doi:10.1152/jn.1975.38.4.871. 808592

[47] Andersen RA, Buneo CA. Intentional maps in posterior parietal cortex. Annu Rev Neurosci 25: 189–220, 2002. doi:10.1146/annurev.neuro.25.112701.142922. 12052908

[48] Rozzi S, Ferrari PF, Bonini L, Rizzolatti G, Fogassi L. Functional organization of inferior parietal lobule convexity in the macaque monkey: electrophysiological characterization of motor, sensory and mirror responses and their correlation with cytoarchitectonic areas. Eur J Neurosci 28: 1569–1588, 2008. doi:10.1111/j.1460-9568.2008.06395.x. 18691325

[49] Jiang HH, Hu YZ, Wang JH, Ma YY, Hu XT. Visuospatial properties of caudal area 7b in *Macaca fascicularis*. Dongwuxue Yanjiu 34: E50–E61, 2013. doi:10.3724/SP.J.1141.2013.E02E50. 23572367

[50] Vierck CJ, Cooper BY, Ritz LA, Greenspan JD. Inference of pain sensitivity from complex behaviors of laboratory animals. In: Issues in Pain Measurement, edited by Chapman CR, Loeser JD. New York: Raven Press, 1989, p. 93–115.

[51] Chudler EH, Sugiyama K, Dong WK. Nociceptive responses in the neostriatum and globus pallidus of the anesthetized rat. J Neurophysiol 69: 1890–1903, 1993. doi:10.1152/jn.1993.69.6.1890. 8350129

[52] Chudler EH, Dong WK. The role of the basal ganglia in nociception and pain. Pain 60: 3–38, 1995. doi:10.1016/0304-3959(94)00172-B. 7715939

[53] Chudler EH, Sugiyama K, Dong WK. Multisensory convergence and integration in the neostriatum and globus pallidus of the rat. Brain Res 674: 33–45, 1995. doi:10.1016/0006-8993(94)01427-J. 7773693

[54] Sugiyama K, Dong WK, Chudler EH. A simplified method for manufacturing glass-insulated metal microelectrodes. J Neurosci Methods 53: 73–80, 1994. doi:10.1016/0165-0270(94)90146-5. 7990516

[55] Dong WK, Shiwaku T, Kawakami Y, Chudler EH. Static and dynamic responses of periodontal ligament mechanoreceptors and intradental mechanoreceptors. J Neurophysiol 69: 1567–1582, 1993. doi:10.1152/jn.1993.69.5.1567. 8389830

[56] Iannetti GD, Mouraux A. From the neuromatrix to the pain matrix (and back). Exp Brain Res 205: 1–12, 2010. doi:10.1007/s00221-010-2340-1. 20607220

[57] Legrain V, Iannetti GD, Plaghki L, Mouraux A. The pain matrix reloaded: a salience detection system for the body. Prog Neurobiol 93: 111–124, 2011. doi:10.1016/j.pneurobio.2010.10.005. 21040755

[58] Morrison I, Perini I, Dunham J. Facets and mechanisms of adaptive pain behavior: predictive regulation and action. Front Hum Neurosci 7: 755, 2013. doi:10.3389/fnhum.2013.00755. 24348358 PMC3842910

[59] Mouraux A, Iannetti GD. The search for pain biomarkers in the human brain. Brain 141: 3290–3307, 2018. doi:10.1093/brain/awy281. 30462175 PMC6262221

[60] de Vignemont F. Expecting pain. Synthese 202: 156–173, 2023. doi:10.1007/s11229-023-04394-x.

[61] Longo MR, Iannetti GD, Mancini F, Driver J, Haggard P. Linking pain and the body: neural correlates of visually induced analgesia. J Neurosci 32: 2601–2607, 2012. doi:10.1523/JNEUROSCI.4031-11.2012. 22357844 PMC6621879

[62] Casey KL, Minoshima S, Morrow TJ, Koeppe RA. Comparison of human cerebral activation pattern during cutaneous warmth, heat pain, and deep cold pain. J Neurophysiol 76: 571–581, 1996. doi:10.1152/jn.1996.76.1.571. 8836245

[63] Derbyshire SW, Jones AK, Gyulai F, Clark S, Townsend D, Firestone LL. Pain processing during three levels of noxious stimulation produces differential patterns of central activity. Pain 73: 431–445, 1997. doi:10.1016/S0304-3959(97)00138-3. 9469535

[64] Svensson P, Minoshima S, Beydoun A, Morrow TJ, Casey KL. Cerebral processing of acute skin and muscle pain in humans. J Neurophysiol 78: 450–460, 1997. doi:10.1152/jn.1997.78.1.450. 9242293

[65] Davis KD, Kwan CL, Crawley AP, Mikulis DJ. Functional MRI study of thalamic and cortical activations evoked by cutaneous heat, cold, and tactile stimuli. J Neurophysiol 80: 1533–1546, 1998. doi:10.1152/jn.1998.80.3.1533. 9744957

[66] Coghill RC, Sang CN, Maisog JM, Iadarola MJ. Pain intensity processing within the human brain: a bilateral, distributed mechanism. J Neurophysiol 82: 1934–1943, 1999. doi:10.1152/jn.1999.82.4.1934. 10515983

[67] Apkarian AV, Darbar A, Krauss BR, Gelnar PA, Szeverenyi NM. Differentiating cortical areas related to pain perception from stimulus identification: temporal analysis of fMRI activity. J Neurophysiol 81: 2956–2963, 1999. doi:10.1152/jn.1999.81.6.2956. 10368412

[68] Coghill RC, Gilron I, Iadarola MJ. Hemispheric lateralization of somatosensory processing. J Neurophysiol 85: 2602–2612, 2001. doi:10.1152/jn.2001.85.6.2602. 11387404

[69] Hofbauer RK, Rainville P, Duncan GH, Bushnell MC. Cortical representation of the sensory dimension of pain. J Neurophysiol 86: 402–411, 2001. doi:10.1152/jn.2001.86.1.402. 11431520

[70] Chen JI, Ha B, Bushnell MC, Pike B, Duncan GH. Differentiating noxious- and innocuous-related activation of human somatosensory cortices using temporal analysis of fMRI. J Neurophysiol 88: 464–474, 2002. doi:10.1152/jn.2002.88.1.464. 12091568

[71] Kong J, White NS, Kwong KK, Vangel MG, Rosman IS, Gracely RH, Gollub RL. Using fMRI to dissociate sensory encoding from cognitive evaluation of heat pain intensity. Hum Brain Mapp 27: 715–721, 2006. doi:10.1002/hbm.20213. 16342273 PMC6871429

[72] Symonds LL, Gordon NS, Bixby JC, Mande MM. Right-lateralized pain processing in the human cortex: an FMRI study. J Neurophysiol 95: 3823–3830, 2006. doi:10.1152/jn.01162.2005. 16554508

[73] Albanese MC, Duerden EG, Rainville P, Duncan GH. Memory traces of pain in human cortex. J Neurosci 27: 4612–4620, 2007. doi:10.1523/JNEUROSCI.0695-07.2007. 17460074 PMC6673011

[74] Kong J, Gollub RL, Polich G, Kirsch I, Laviolette P, Vangel M, Rosen B, Kaptchuk TJ. A functional magnetic resonance imaging study on the neural mechanisms of hyperalgesic nocebo effect. J Neurosci 28: 13354–13362, 2008. doi:10.1523/JNEUROSCI.2944-08.2008. 19052227 PMC2649754

[75] Baumgärtner U, Iannetti GD, Zambreanu L, Stoeter P, Treede RD, Tracey I. Multiple somatotopic representations of heat and mechanical pain in the operculo-insular cortex: a high-resolution fMRI study. J Neurophysiol 104: 2863–2872, 2010. doi:10.1152/jn.00253.2010. 20739597 PMC2997041

[76] Mouraux A, Diukova A, Lee MC, Wise RG, Iannetti GD. A multisensory investigation of the functional significance of the “pain matrix”. Neuroimage 54: 2237–2249, 2011. doi:10.1016/j.neuroimage.2010.09.084. 20932917

[77] Chen LM, Dillenburger BC, Wang F, Tang CH. Differential fMRI activation to noxious heat and tactile stimuli in parasylvian areas of new world monkeys. Pain 153: 158–169, 2012. doi:10.1016/j.pain.2011.10.006. 22115923 PMC3245780

[78] Mazzola L, Faillenot I, Barral FG, Mauguière F, Peyron R. Spatial segregation of somato-sensory and pain activations in the human operculo-insular cortex. Neuroimage 60: 409–418, 2012. doi:10.1016/j.neuroimage.2011.12.072. 22245639

[79] Moulton EA, Pendse G, Becerra LR, Borsook D. BOLD responses in somatosensory cortices better reflect heat sensation than pain. J Neurosci 32: 6024–6031, 2012. doi:10.1523/JNEUROSCI.0006-12.2012. 22539862 PMC3347471

[80] zu Eulenburg P, Baumgärtner U, Treede RD, Dieterich M. Interoceptive and multimodal functions of the operculo-insular cortex: tactile, nociceptive and vestibular representations. Neuroimage 83: 75–86, 2013. doi:10.1016/j.neuroimage.2013.06.057. 23800791

[81] Favilla S, Huber A, Pagnoni G, Lui F, Facchin P, Cocchi M, Baraldi P, Porro CA. Ranking brain areas encoding the perceived level of pain from fMRI data. Neuroimage 90: 153–162, 2014. doi:10.1016/j.neuroimage.2014.01.001. 24418504

[82] Wu R, Wang F, Yang PF, Chen LM. High-resolution functional MRI identified distinct global intrinsic functional networks of nociceptive posterior insula and S2 regions in squirrel monkey brain. Neuroimage 155: 147–158, 2017. doi:10.1016/j.neuroimage.2017.04.067. 28461059 PMC6104393

[83] Horing B, Sprenger C, Büchel C. The parietal operculum preferentially encodes heat pain and not salience. PLoS Biol 17: e3000205, 2019. doi:10.1371/journal.pbio.3000205. 31404058 PMC6705876

[84] Liang M, Su Q, Mouraux A, Iannetti GD. Spatial patterns of brain activity preferentially reflecting transient pain and stimulus intensity. Cereb Cortex 29: 2211–2227, 2019. doi:10.1093/cercor/bhz026. 30844052 PMC6458907

[85] Su Q, Qin W, Yang QQ, Yu CS, Qian TY, Mouraux A, Iannetti GD, Liang M. Brain regions preferentially responding to transient and iso-intense painful or tactile stimuli. Neuroimage 192: 52–65, 2019. doi:10.1016/j.neuroimage.2019.01.039. 30669009 PMC6503155

[86] Fauchon C, Meunier D, Faillenot I, Pomares FB, Bastuji H, Garcia-Larrea L, Peyron R. The modular organization of pain brain networks: an fMRI graph analysis informed by intracranial EEG. Cereb Cortex Commun 1: tgaa088, 2020. doi:10.1093/texcom/tgaa088. 34296144 PMC8152828

[87] Song Y, Su Q, Yang Q, Zhao R, Yin G, Qin W, Iannetti GD, Yu C, Liang M. Feedforward and feedback pathways of nociceptive and tactile processing in human somatosensory system: a study of dynamic causal modeling of fMRI data. Neuroimage 234: 117957, 2021. doi:10.1016/j.neuroimage.2021.117957. 33744457

[88] Wiech K, Jbabdi S, Lin CS, Andersson J, Tracey I. Differential structural and resting state connectivity between insular subdivisions and other pain-related brain regions. Pain 155: 2047–2055, 2014. doi:10.1016/j.pain.2014.07.009. 25047781 PMC4220010

[89] Segerdahl AR, Mezue M, Okell TW, Farrar JT, Tracey I. The dorsal posterior insula subserves a fundamental role in human pain. Nat Neurosci 18: 499–500, 2015 [Erratum in Nat Neurosci 18: 1861, 2015]. doi:10.1038/nn.3969. 25751532 PMC6783299

[90] Eickhoff SB, Schleicher A, Zilles K, Amunts K. The human parietal operculum. I. Cytoarchitectonic mapping of subdivisions. Cereb Cortex 16: 254–267, 2006. doi:10.1093/cercor/bhi105. 15888607

[91] Eickhoff SB, Amunts K, Mohlberg H, Zilles K. The human parietal operculum. II. Stereotaxic maps and correlation with functional imaging results. Cereb Cortex 16: 268–279, 2006. doi:10.1093/cercor/bhi106. 15888606

[92] Eickhoff SB, Grefkes C, Zilles K, Fink GR. The somatotopic organization of cytoarchitectonic areas on the human parietal operculum. Cereb Cortex 17: 1800–1811, 2007. doi:10.1093/cercor/bhl090. 17032710

[93] Whitsel BL, Petrucelli LM, Werner G. Symmetry and connectivity in the map of the body surface in somatosensory area II of primates. J Neurophysiol 32: 170–183, 1969. doi:10.1152/jn.1969.32.2.170. 4975532

[94] Chudler EH, Dong WK, Kawakami Y. Tooth pulp-evoked potentials in the monkey: cortical surface and intracortical distribution. Pain 22: 221–233, 1985. doi:10.1016/0304-3959(85)90022-3. 4034222

[95] Chudler EH, Dong WK, Kawakami Y. Cortical nociceptive responses and behavioral correlates in the monkey. Brain Res 397: 47–60, 1986. doi:10.1016/0006-8993(86)91368-5. 3801865

[96] Burton H, Sinclair RJ, Hong SY, Pruett JR Jr, Whang KC. Tactile-spatial and cross-modal attention effects in the second somatosensory and 7b cortical areas of rhesus monkeys. Somatosens Mot Res 14: 237–267, 1997. doi:10.1080/08990229770971. 9443366

[97] Lenz FA, Rios M, Chau D, Krauss GL, Zirh TA, Lesser RP. Painful stimuli evoke potentials recorded from the parasylvian cortex in humans. J Neurophysiol 80: 2077–2088, 1998. doi:10.1152/jn.1998.80.4.2077. 9772262

[98] Frot M, Mauguière F. Timing and spatial distribution of somatosensory responses recorded in the upper bank of the sylvian fissure (SII area) in humans. Cereb Cortex 9: 854–863, 1999. doi:10.1093/cercor/9.8.854. 10601004

[99] Frot M, Rambaud L, Guénot M, Mauguière F. Intracortical recordings of early pain-related CO_2_-laser evoked potentials in the human second somatosensory (SII) area. Clin Neurophysiol 110: 133–145, 1999. doi:10.1016/S0168-5597(98)00054-9. 10348332

[100] Frot M, Magnin M, Mauguière F, Garcia-Larrea L. Human SII and posterior insula differently encode thermal laser stimuli. Cereb Cortex 17: 610–620, 2007. doi:10.1093/cercor/bhk007. 16614165

[101] Timmermann L, Ploner M, Haucke K, Schmitz F, Baltissen R, Schnitzler A. Differential coding of pain intensity in the human primary and secondary somatosensory cortex. J Neurophysiol 86: 1499–1503, 2001. doi:10.1152/jn.2001.86.3.1499. 11535693

[102] Nakamura Y, Paur R, Zimmermann R, Bromm B. Attentional modulation of human pain processing in the secondary somatosensory cortex: a magnetoencephalographic study. Neurosci Lett 328: 29–32, 2002. doi:10.1016/S0304-3940(02)00447-0. 12123852

[103] Vogel H, Port JD, Lenz FA, Solaiyappan M, Krauss G, Treede RD. Dipole source analysis of laser-evoked subdural potentials recorded from parasylvian cortex in humans. J Neurophysiol 89: 3051–3060, 2003. doi:10.1152/jn.00772.2002. 12783950

[104] Ohara S, Crone NE, Weiss N, Treede RD, Lenz FA. Amplitudes of laser evoked potential recorded from primary somatosensory, parasylvian and medial frontal cortex are graded with stimulus intensity. Pain 110: 318–328, 2004. doi:10.1016/j.pain.2004.04.009. 15275782

[105] Torquati K, Pizzella V, Babiloni C, Del Gratta C, Della Penna S, Ferretti A, Franciotti R, Rossini PM, Romani GL. Nociceptive and non-nociceptive sub-regions in the human secondary somatosensory cortex: an MEG study using fMRI constraints. Neuroimage 26: 48–56, 2005. doi:10.1016/j.neuroimage.2005.01.012. 15862204

[106] Mazzola L, Isnard J, Mauguière F. Somatosensory and pain responses to stimulation of the second somatosensory area (SII) in humans. A comparison with SI and insular responses. Cereb Cortex 16: 960–968, 2006. doi:10.1093/cercor/bhj038. 16177270

[107] Hinkley LB, Krubitzer LA, Nagarajan SS, Disbrow EA. Sensorimotor integration in S2, PV, and parietal rostroventral areas of the human sylvian fissure. J Neurophysiol 97: 1288–1297, 2007. doi:10.1152/jn.00733.2006. 17122318 PMC4060608

[108] Frot M, Mauguière F, Magnin M, Garcia-Larrea L. Parallel processing of nociceptive A-delta inputs in SII and midcingulate cortex in humans. J Neurosci 28: 944–952, 2008. doi:10.1523/JNEUROSCI.2934-07.2008. 18216202 PMC6670999

[109] Garcia-Larrea L, Perchet C, Creac’h C, Convers P, Peyron R, Laurent B, Mauguière F, Magnin M. Operculo-insular pain (parasylvian pain): a distinct central pain syndrome. Brain 133: 2528–2539, 2010. doi:10.1093/brain/awq220. 20724291

[110] Bastuji H, Frot M, Perchet C, Magnin M, Garcia-Larrea L. Pain networks from the inside: spatiotemporal analysis of brain responses leading from nociception to conscious perception. Hum Brain Mapp 37: 4301–4315, 2016. doi:10.1002/hbm.23310. 27391083 PMC6867521

[111] Ye X, Yang PF, Liu Q, Dillenburger BD, Friedman RM, Chen LM. A thermal nociceptive patch in the S2 cortex of nonhuman primates: a combined functional magnetic resonance imaging and electrophysiology study. Pain 162: 2705–2716, 2021. doi:10.1097/j.pain.0000000000002247. 33945242 PMC8380756

[112] Gardner EP, Babu KS, Reitzen SD, Ghosh S, Brown AS, Chen J, Hall AL, Herzlinger MD, Kohlenstein JB, Ro JY. Neurophysiology of prehension. I. Posterior parietal cortex and object-oriented hand behaviors. J Neurophysiol 97: 387–406, 2007. doi:10.1152/jn.00558.2006. 16971679 PMC2868366

[113] Sathian K. Analysis of haptic information in the cerebral cortex. J Neurophysiol 116: 1795–1806, 2016. doi:10.1152/jn.00546.2015. 27440247 PMC5144710

[114] Nomi JS, Schettini E, Broce I, Dick AS, Uddin LQ. Structural connections of functionally defined human insular subdivisions. Cereb Cortex 28: 3445–3456, 2018. doi:10.1093/cercor/bhx211. 28968768 PMC6132280

[115] Delhaye BP, Long KH, Bensmaia SJ. Neural basis of touch and proprioception in primate cortex. Compr Physiol 8: 1575–1602, 2018. doi:10.1002/cphy.c170033. 30215864 PMC6330897

[116] Legrain V, Filbrich L, Vanderclausen C. Letter on the pain of blind people for the use of those who can see their pain. Pain 164: 1451–1456, 2023. doi:10.1097/j.pain.0000000000002862. 36728808

[117] De Paepe AL, Crombez G, Spence C, Legrain V. Mapping nociceptive stimuli in a peripersonal frame of reference: evidence from a temporal order judgment task. Neuropsychologia 56: 219–228, 2014. doi:10.1016/j.neuropsychologia.2014.01.016. 24486423

[118] De Paepe AL, Crombez G, Legrain V. From a somatotopic to a spatiotopic frame of reference for the localization of nociceptive stimuli. PLoS One 10: e0137120, 2015. doi:10.1371/journal.pone.0137120. 26317671 PMC4552762

[119] Bufacchi RJ, Iannetti GD. Gravitational cues modulate the shape of defensive peripersonal space. Curr Biol 26: R1133–R1134, 2016. doi:10.1016/j.cub.2016.09.025. 27825445 PMC5106387

[120] Somervail R, Bufacchi RJ, Guo Y, Kilintari M, Novembre G, Swapp D, Steed A, Iannetti GD. Movement of environmental threats modifies the relevance of the defensive eye-blink in a spatially-tuned manner. Sci Rep 9: 3661, 2019. doi:10.1038/s41598-019-40075-x. 30842481 PMC6403335

[121] Bisio A, Garbarini F, Biggio M, Fossataro C, Ruggeri P, Bove M. Dynamic shaping of the defensive peripersonal space through predictive motor mechanisms: when the “near” becomes “far”. J Neurosci 37: 2415–2424, 2017. doi:10.1523/JNEUROSCI.0371-16.2016. 28154151 PMC6596839

[122] Bufacchi RJ. Approaching threatening stimuli cause an expansion of defensive peripersonal space. J Neurophysiol 118: 1927–1930, 2017. doi:10.1152/jn.00316.2017. 28539400 PMC5626904

[123] Gallace A, Torta DM, Moseley GL, Iannetti GD. The analgesic effect of crossing the arms. Pain 152: 1418–1423, 2011. doi:10.1016/j.pain.2011.02.029. 21440992

[124] Longo MR, Betti V, Aglioti SM, Haggard P. Visually induced analgesia: seeing the body reduces pain. J Neurosci 29: 12125–12130, 2009. doi:10.1523/JNEUROSCI.3072-09.2009. 19793970 PMC6666129

[125] Sambo CF, Torta DM, Gallace A, Liang M, Moseley GL, Iannetti GD. The temporal order judgement of tactile and nociceptive stimuli is impaired by crossing the hands over the body midline. Pain 154: 242–247, 2013. doi:10.1016/j.pain.2012.10.010. 23200703

[126] Torta DM, Legrain V, Mouraux A. Looking at the hand modulates the brain responses to nociceptive and non-nociceptive somatosensory stimuli but does not necessarily modulate their perception. Psychophysiology 52: 1010–1018, 2015. doi:10.1111/psyp.12439. 25917217 PMC5338730

[127] Kobatake E, Tanaka K. Neuronal selectivities to complex object features in the ventral visual pathway of the macaque cerebral cortex. J Neurophysiol 71: 856–867, 1994. doi:10.1152/jn.1994.71.3.856. 8201425

[128] Mesulam MM. Spatial attention and neglect: parietal, frontal and cingulate contributions to the mental representation and attentional targeting of salient extrapersonal events. Philos Trans R Soc Lond B Biol Sci 354: 1325–1346, 1999 [Erratum in Philos Trans R Soc Lond B Biol Sci 354: 2083, 1999]. doi:10.1098/rstb.1999.0482. 10466154 PMC1692628

[129] Quevedo AS, Coghill RC. Attentional modulation of spatial integration of pain: evidence for dynamic spatial tuning. J Neurosci 27: 11635–11640, 2007. doi:10.1523/JNEUROSCI.3356-07.2007. 17959806 PMC6673211

[130] Motter BC, Mountcastle VB. The functional properties of the light-sensitive neurons of the posterior parietal cortex studied in waking monkeys: foveal sparing and opponent vector organization. J Neurosci 1: 3–26, 1981. doi:10.1523/JNEUROSCI.01-01-00003.1981. 7346556 PMC6564161

[131] Forss N, Raij TT, Seppä M, Hari R. Common cortical network for first and second pain. Neuroimage 24: 132–142, 2005. doi:10.1016/j.neuroimage.2004.09.032. 15588604

[132] Nakata H, Tamura Y, Sakamoto K, Akatsuka K, Hirai M, Inui K, Hoshiyama M, Saitoh Y, Yamamoto T, Katayama Y, Kakigi R. Evoked magnetic fields following noxious laser stimulation of the thigh in humans. Neuroimage 42: 858–868, 2008. doi:10.1016/j.neuroimage.2008.05.017. 18585060

[133] Manfron L, Filbrich L, Nijs E, Mouraux A, Legrain V. Investigating perceptual simultaneity between nociceptive and visual stimuli by means of temporal order judgments. Neurosci Lett 735: 135156, 2020. doi:10.1016/j.neulet.2020.135156. 32574797

[134] Manfron L, Filbrich L, Molitor V, Farnè A, Mouraux A, Legrain V. Perceptual simultaneity between nociceptive and visual stimuli depends on their spatial congruence. Exp Brain Res 241: 1785–1796, 2023. doi:10.1007/s00221-023-06637-2. 37222776

[135] Meredith MA, Stein BE. Visual, auditory, and somatosensory convergence on cells in superior colliculus results in multisensory integration. J Neurophysiol 56: 640–662, 1986. doi:10.1152/jn.1986.56.3.640. 3537225

[136] Wallace MT, Wilkinson LK, Stein BE. Representation and integration of multiple sensory inputs in primate superior colliculus. J Neurophysiol 76: 1246–1266, 1996. doi:10.1152/jn.1996.76.2.1246. 8871234

[137] Wagman IH, Price DD. Responses of dorsal horn cells of *M. mulatta* to cutaneous and sural nerve A and C fiber stimuli. J Neurophysiol 32: 803–817, 1969. doi:10.1152/jn.1969.32.6.803. 4981517

[138] Dong WK, Ryu H, Wagman IH. Nociceptive responses of neurons in medial thalamus and their relationship to spinothalamic pathways. J Neurophysiol 41: 1592–1613, 1978. doi:10.1152/jn.1978.41.6.1592. 731292

[139] Thomson AM. Facilitation, augmentation and potentiation at central synapses. Trends Neurosci 23: 305–312, 2000. doi:10.1016/S0166-2236(00)01580-0. 10856940

[140] Thomson AM. Molecular frequency filters at central synapses. Prog Neurobiol 62: 159–196, 2000. doi:10.1016/S0301-0082(00)00008-3. 10828382

[141] Zucker RS, Regehr WG. Short-term synaptic plasticity. Annu Rev Physiol 64: 355–405, 2002. doi:10.1146/annurev.physiol.64.092501.114547. 11826273

[142] Kaeser PS, Regehr WG. Molecular mechanisms for synchronous, asynchronous, and spontaneous neurotransmitter release. Annu Rev Physiol 76: 333–363, 2014. doi:10.1146/annurev-physiol-021113-170338. 24274737 PMC4503208

[143] Jackman SL, Regehr WG. The mechanisms and functions of synaptic facilitation. Neuron 94: 447–464, 2017. doi:10.1016/j.neuron.2017.02.047. 28472650 PMC5865607

[144] Robinson CJ, Burton H. Organization of somatosensory receptive fields in cortical areas 7b, retroinsula, postauditory and granular insula of *M. fascicularis*. J Comp Neurol 192: 69–92, 1980. doi:10.1002/cne.901920105. 7410614

[145] Neal JW, Pearson RC, Powell TP. The cortico-cortical connections of area 7b, PF, in the parietal lobe of the monkey. Brain Res 419: 341–346, 1987. doi:10.1016/0006-8993(87)90605-6. 2445426

[146] Neal JW, Pearson RC, Powell TP. The ipsilateral cortico-cortical connections of area 7b, PF, in the parietal and temporal lobes of the monkey. Brain Res 524: 119–132, 1990. doi:10.1016/0006-8993(90)90500-B. 1698108

[147] Neal JW, Pearson RC, Powell TP. The ipsilateral corticocortical connections of area 7 with the frontal lobe in the monkey. Brain Res 509: 31–40, 1990. doi:10.1016/0006-8993(90)90305-U. 1689604

[148] Goldman-Rakic PS. Topography of cognition: parallel distributed networks in primate association cortex. Annu Rev Neurosci 11: 137–156, 1988. doi:10.1146/annurev.ne.11.030188.001033. 3284439

[149] Friedman DP, Murray EA, O’Neill JB, Mishkin M. Cortical connections of the somatosensory fields of the lateral sulcus of macaques: evidence for a corticolimbic pathway for touch. J Comp Neurol 252: 323–347, 1986. doi:10.1002/cne.902520304. 3793980

[150] Graziano MS, Hu XT, Gross CG. Visuospatial properties of ventral premotor cortex. J Neurophysiol 77: 2268–2292, 1997. doi:10.1152/jn.1997.77.5.2268. 9163357

[151] Cooke DF, Graziano MS. Defensive movements evoked by air puff in monkeys. J Neurophysiol 90: 3317–3329, 2003. doi:10.1152/jn.00513.2003. 12801896

[152] Graziano MS, Cooke DF. Parieto-frontal interactions, personal space, and defensive behavior. Neuropsychologia 44: 2621–2635, 2006. doi:10.1016/j.neuropsychologia.2005.09.011. 17128446

[153] Orban GA, Sepe A, Bonini L. Parietal maps of visual signals for bodily action planning. Brain Struct Funct 226: 2967–2988, 2021. doi:10.1007/s00429-021-02378-6. 34508272 PMC8541987

[154] Goldring AB, Cooke DF, Pineda CR, Recanzone GH, Krubitzer LA. Functional characterization of the fronto-parietal reaching and grasping network: reversible deactivation of M1 and areas 2, 5, and 7b in awake behaving monkeys. J Neurophysiol 127: 1363–1387, 2022. doi:10.1152/jn.00279.2021. 35417261 PMC9109808

[155] Morrison I, Tipper SP, Fenton-Adams WL, Bach P. “Feeling” others’ painful actions: the sensorimotor integration of pain and action information. Hum Brain Mapp 34: 1982–1998, 2013. doi:10.1002/hbm.22040. 22451259 PMC3807605

[156] Perini I, Bergstrand S, Morrison I. Where pain meets action in the human brain. J Neurosci 33: 15930–15939, 2013. doi:10.1523/JNEUROSCI.3135-12.2013. 24089498 PMC6618478

[157] Koppel L, Novembre G, Kämpe R, Savallampi M, Morrison I. Prediction and action in cortical pain processing. Cereb Cortex 33: 794–810, 2023. doi:10.1093/cercor/bhac102. 35289367 PMC9890457

